# Baseline Assessment of Evidence-Based Intrapartum Care Practices in Medical Schools in 3 States in India: A Mixed-Methods Study

**DOI:** 10.9745/GHSP-D-21-00590

**Published:** 2022-04-28

**Authors:** Kirti Iyengar, Madhu Gupta, Swarnika Pal, Kiranjit Kaur, Neena Singla, Madhur Verma, Anchal Dhiman, Rimpi Singla, Minakshi Rohilla, Vanita Suri, Neelam Aggarwal, Tarundeep Singh, Poonam Goel, N. K. Goel, Reena Pant, Kusum Lata Gaur, Hanslata Gehlot, Indra Bhati, Manoj Verma, Sudesh Agarwal, Rekha Acharya, Keerti Singh, Madhubala Chauhan, Radha Rastogi, Renu Bedi, Poornima Pancholi, Bipin Nayak, Bhavesh Modi, Kanaklata Nakum, Atul Trivedi, Shonali Aggarwal, Sangita Patel

**Affiliations:** a United Nations Population Fund, New Delhi, India.; b Postgraduate Institute of Medical Education and Research (PGIMER), Chandigarh, India.; c Seth GS Medical College & KEM Hospital, Mumbai, India.; d All India Institute Medical Science, Bathinda, Punjab, India.; e Government Medical College and Hospital, Chandigarh, India.; f Swai Maan Singh Medical College, Jaipur, Rajasthan, India.; g Dr. Sampurnanand Medical College and Hospital, Jodhpur, Rajasthan, India.; h Sardar Patel Medical College and PBM Hospital, Bikaner Rajasthan, India.; i Rabindranath Tagore Medical College and Hospital, Udaipur, Rajasthan, India.; j Jawaharlal Nehru Medical College and Hospital, Ajmer Rajasthan, India.; k GMERS Medical College and Hospital, Gandhinagar, Gujarat, India.; l Government Medical College and Hospital, Bhavnagar, Gujarat, India.; m Government Medical College and Hospital, Baroda, Gujarat, India.

## Abstract

**Introduction:**

Implementation research with pre- and post-comparison was planned to improve the quality of evidence-based intrapartum care services in Indian medical schools. We present the baseline study results to assess the status of adherence to intrapartum evidence-based practices (IP-EBP) in study schools in 3 states in India and the perception of the faculty.

**Methods:**

A concurrent mixed-methods approach was used to conduct the baseline assessment in 9 medical schools in Rajasthan, Gujarat, and Union Territory from October 2018 to June 2019. IP-EBP among pregnant women in uncomplicated first (n=135), second (n=120), and third stage (n=120) of labor were observed using a predesigned, pretested checklist quantitatively. We conducted in-depth interviews with 33 obstetrics and gynecology faculty to understand their perceptions of intrapartum practices. Quantitative data were analyzed using SPSS (version 22). COM-B (Capability, Opportunity, and Motivation Behavior) model was used to understand the behaviors, and thematic analysis was done for the qualitative data.

**Findings:**

Unindicated augmentation of labor was done in 64.4%, fundal pressure applied in 50.8%, episiotomy done in 58.3%, and delivery in lithotomy position was performed in 86.7% of women in labor.

**Conclusions:**

Intrapartum practices that are not recommended were routinely practiced in the study medical schools due to a lack of staff awareness of evidence-based practices and incorrect beliefs about their impact.

## Abstract (precis)

The study findings have identified significant gaps between current intrapartum care practices and recommended national and international guidelines in the medical schools of 3 states in India.

## Teaser

**Key Findings:**

- In the study medical schools, we observed providers using several harmful or unnecessary practices on pregnant women in labor, including routine pubic shaving, enema on admission, routine episiotomy, application of fundal pressure, delivery in the lithotomy position, and unindicated augmentation.
- Barriers to adherence to the recommended evidence-based intrapartum practices included fear of perineal tear/injury to the baby in different birthing positions; lack of physical space, resources, and time; and outdated knowledge and beliefs of faculty and labor room staff.

**Key Implications:**

- Advocacy efforts are needed with medical councils and state departments governing medical education to strengthen training and upscale skills and provide resources to support positive childbirth experiences for laboring women.
- Capacity building of medical educators and staff through organizing continued medical education, skill development, teacher's training programs, and refresher courses on evidence-based practices should be done. Medical schools' curricula should include lectures and practical sessions on evidence-based intrapartum care practices, neonatal care, and respectful maternity care in medical schools.
- Implementation research should be carried out in the medical schools to develop optimum need-based interventions to bring about sustainable changes in medical school practices as per the recommended evidence-based guidelines.

## INTRODUCTION

Every year, the majority of 140 million births occur globally without risk factors for complications.^1^ However, more than 70% of maternal deaths globally and 80% in India are due to direct obstetric labor and delivery complications.^2^ It is estimated that more than 40% of stillbirths and 40% of neonatal deaths occur on the day of the delivery in India.^3^ Effective interventions during this critical period can reduce maternal deaths significantly.^4^

According to the World Health Organization (WHO) Quality of Care Framework for maternal and newborn health, evidence-based care practice is a crucial quality-of-care component.^1^ WHO's recommendations on intrapartum care for a positive childbirth experience are based on the results of Cochrane's systematic reviews with Grading of Recommendations Assessment, Development and Evaluation (GRADE) and Confidence in the Evidence from Reviews of Qualitative research (CERQual) approaches for quantitative and qualitative evidence, respectively. WHO issued 56 recommendations on intrapartum care—26 new and 30 were integrated from existing guidelines. Some of the essential recommended intrapartum care practices for a positive childbirth experience based on Cochrane's systematic review included encouraging mobility during labor (25 trials), having a birth companion (26 trials), offering the position of choice for delivery (5 trials), delayed cord clamping (3 trials), performing skin-to-skin contact of the baby with the mother (4 trials), initiating breastfeeding early (4 trials), and promoting respectful maternity care (5 studies). Practices not recommended for a positive birth experience included pubic shaving (3 trials), using enemas (4 trials), augmenting labor routinely (3 trials), using the lithotomy position for delivery (5 trials), applying fundal pressure (9 trials), and performing routine episiotomy (12 trials).^1^

Evidence exists that using interventions in the first stage of labor that are not recommended (e.g., enema, amniotomy, restricted mobility, continuous electronic fetal monitoring, and routine episiotomy on delivery) had an adverse impact on the birth process in terms of increased cesarean delivery rate due to fetal distress, perineum abrasions at birth, placenta split-up duration longer than 30 minutes, and complications like manual removal of placenta. These interventions also adversely affected newborn health in terms of shoulder dystocia, higher intensive care need, and trauma to the newborn's arms, face, and back.^5^ Increased interventions in the first stage of labor increased the need for interventions (fundal pressure and episiotomy) in the second stage. Litrop et al. reported that unindicated augmentation of labor led to an increased risk of adverse perinatal outcomes such as bag-and-mask ventilation, low Apgar score, and neonatal death in 12 public hospitals in Nepal.^6^ Thus, adherence to evidence-based intrapartum care practices is essential for achieving better maternal and perinatal outcomes.^7^

In India, the proportion of institutional deliveries in public health facilities rapidly increased from 38.7% (2005–2006) to 78.9% (2015–2016)^8^ due to the implementation of the conditional cash transfer schemes^9^ and the availability of free transport services as part of National Rural Health Mission in 2005. Although these interventions led to an increase in the number of women being admitted to the labor rooms for delivery, the corresponding pace of strengthening human resources, infrastructure, and capacity building of the labor room staff was slow. This mismatch in demand and supply resulted in poor quality of care in terms of adherence to evidence-based intrapartum care practices and respectful maternal care provided to the laboring women.^10^^,^^11^

Singh et al. reported that despite the increase in institutional deliveries, the maternal mortality ratio did not decline considerably.^12^ Some studies have reported an increased maternal mortality ratio in tertiary care institutes due to delivery overload and poor quality of services rendered after delivery.^10^^,^^13^^,^^14^ A study to improve the quality of intrapartum services in Rajasthan showed that the dorsal position during delivery was used in all facilities and augmentation of labor and routine episiotomy for primigravids was routinely used in 93% and 77% of facilities, which affected newborn health and survival.^15^^,^^16^ Another study from Rajasthan reported severe gaps in the quality of intrapartum care with the widespread use of unnecessary practices such as augmentation of labor, application of strong fundal pressure, and inadequate use of beneficial practices.^17^ A study in the city of Bursa reported induction of labor in 60.0% of cases, fundal pressure application in 30.6%, and episiotomy in 63.7% of cases.^18^ Birthing position of choice was not offered to 92% of women, and additionally, physical violence and verbal abuse, while she was bearing down, was observed in a study in Uttar Pradesh.^19^

Interventions in India have increased the institutional delivery rate but have not resulted in improved quality of care.

The intrapartum care practices followed in the medical schools are a benchmark for the students and determine their practices in the future. Iyengar et al. highlighted the fact that many doctors in Rajasthan preferred using practices that they had learned and observed in medical schools, and adopting new evidence-based intrapartum practices meant more significant efforts on their part and added to the workload in their already busy schedules.^15^ A study by Gupta et al. highlighted that the training provided to medical students during the undergraduate period is inadequate to equip them to provide future services.^20^ According to a WHO report on evidence-led obstetrics care, most decisions related to intrapartum care during labor and childbirth were still based on personal beliefs, traditions, anecdotes, and clinical observation rather than evidence-based research findings,^21^ and often, strong opinions of care providers regarding specific interventions presented a significant barrier toward changing behaviors.^22^

With this background, we planned to conduct implementation research to improve the adherence to evidence-based intrapartum care practices in medical schools and to improve the quality of related medical teaching so that providers practice improved care during the intrapartum period and medical students have greater sensitivity toward respectful maternity care. We present the baseline assessment findings related to intrapartum evidence-based practices (IP-EBP) in the study medical schools. The baseline evaluation aimed to assess the status of adherence to IP-EBP in the study medical schools and explore the perceptions of medical educators of the department of obstetrics and gynecology regarding the actual implementation of these practices.

## METHODS

### Study Design

We planned to conduct implementation research with pre- and post-comparison. It had 3 phases: (1) baseline assessment for problem scoping; (2) implementation phase to deliver the need-based intervention in consultation with the stakeholders (faculty, doctors, and nurses); and (3) endline assessment phase. Practical approaches included need-based and feasible interventions to increase the sustainability, equity, efficiency, and scale-up of the health intervention.^23^ These interventions were based upon partnerships between the researchers, implementers, and policy makers.

A concurrent mixed methods study design was used to obtain the information at the baseline and endline assessment phases to obtain a comprehensive understanding of the causative factors responsible for the existing intrapartum care practices in the medical schools. The quantitative study documented the actual practices observed, and the qualitative study explored the perception and beliefs of the faculty members about IP-EBP, which contributed to the practices. We present the baseline assessment findings in this article. The quantitative component included observing the first, second, and third stages of uncomplicated labor and delivery in the labor room. The qualitative component included in-depth faculty interviews regarding IP-EBP and family planning services. During the same visit, we conducted direct observations of women in labor, face-to-face interviews with postnatal women, and faculty members. Mixing of the quantitative and qualitative data was done at the interpretation level to infer the explanation of those practices, vis-à-vis the perceptions of the service providers. Mixing the data means integrating, connecting, and supporting the 3 independent data components with one another. Qualitative data are used to understand the quantitative data's pathways or underpinnings.

### Study Area and Health Facilities

We used a 2-stage process to select the states and then the medical schools. United Nations Population Fund (UNFPA) organized a national-level workshop with the Director of Medical Education and Research (DMER) of Indian states to strengthen pre-service evidence-based practices in the medical schools. States were selected purposively based on the following criteria: (1) states volunteering themselves, and (2) states suggested by the funding agency to conduct the study. In the workshop, as per UNFPA India's suggestion, we purposively selected Rajasthan and Gujarat states as UNFPA supported these states to strengthen maternal health and family welfare programs. The DMER of a union territory (UT) volunteered to participate in this study.

In the second stage, a list of all (N=29) government medical schools was made from the selected states (Rajasthan n=21; Gujarat n=7) and UT (n=1). The selection criteria of medical schools included: (1) had 10 or more years of experience in health service delivery and teaching of undergraduates, (2) received ethical approval or permission from the DMER to conduct the study in the medical school as per the local situation, and (3) was a government medical school. These criteria excluded all the private and new government medical schools. We did not use ranking and performance criteria to select the study medical colleges. Based on the inclusion criteria, we selected 5 medical schools in Rajasthan, 3 in Gujarat, and 1 in UT.

According to the National Family Health Survey round 5 (2019–2021), the rate of institutional deliveries in the selected states was among the highest in the country and higher than the national average (88.6%): Rajasthan had 94.9%, Gujarat 94.3%, and UT 96.9%.^5^ Of these institutional deliveries, those delivered in a public health facility were 77% in Rajasthan, 43.3% in Gujarat, and 83.2% in the selected UT. The percentage of institutional deliveries conducted in the study medical schools of all deliveries conducted in public health facilities was estimated to be 8.7%–18.8% in 5 medical schools in Rajasthan, 7.2%–25.1% in 3 medical schools in Gujarat, and 23% in UT.

### Study Period

The baseline assessment was conducted in Gujarat, Rajasthan, and UT from October 2018 to June 2019.

### Quantitative Study

We calculated a sample size of 154 laboring women using the formula to compare the differences in proportion using the sample size calculator given by Fleiss et al. for the quantitative study.^24^ Assuming the proportion of deliveries with at least 3 crucial evidence-based practices (avoiding unindicated augmentation of labor, avoiding a routine episiotomy, using alternate birthing positions for delivery during intrapartum care) 10% at the baseline and 40% at the endline, type I error 5%, power 80%, design effect of 2, and nonresponse rate of 10%, the sample size is estimated at 154. Hence, we observed 155 deliveries (15 in each study medical school) in the baseline assessment. In addition, we calculated a sample size of 135 postnatal women using the formula as estimated for laboring women with a design effect of 1.5. Hence, we interviewed 136 postnatal women in the postnatal ward regarding their experience of labor and delivery in medical schools.

#### Data Collection Method

Data were collected by the independent research team based at the central coordinating institute. The research team from this institute comprised of 1 faculty member (MD in obstetrics and gynecology or community medicine), 1 project officer (MD in community medicine), and a research associate (Master in Public Health). The faculty member from the coordinating institute served primarily to supervise and facilitate the data collection. The faculty members from the study institutions were the coinvestigators, not part of the data collection team at the central coordinating institute. The project officer and research associate mainly collected data. The same team visited all the medical schools; the different faculty members in community medicine or the obstetrics and gynecology department from the coordinating institute visited to supervise and facilitate the data collection. All team members were female. The obstetrics and gynecology faculty in the central coordinating institute trained the project officer and research associate in the labor room practices. The research staff also had experience assisting normal vaginal deliveries as a part of their graduation and post-graduation training.

Observation of deliveries in the labor room of each medical school was done for 3 days for approximately 8 hours in rotation by 2 researchers. Investigators of the respective medical schools facilitated and gave their permission to collect the data. Direct observation of women with uncomplicated pregnancy delivering vaginally was done in the first, second, and third stages of labor till the time woman remained admitted in the labor room. All those women who met the inclusion criteria (i.e., uncomplicated full-term pregnancy in spontaneous labor), were selected consecutively. In case the woman developed complications after inclusion in the study, the observation was stopped, and data were included until she no longer had complications. So, if a woman developed complications during stage 1, observations were recorded for stage 1 only. If the woman was admitted in labor during stage 2 of labor, observations during stages 2 and 3 were recorded, if she met the inclusion criteria. Overall, we observed 155 laboring women. Among these, 100 women were observed in all stages (1, 2, and 3) of labor, 35 in the first stage only, and 20 in the second and third stages. Among the woman observed in the first stage of labor, 2 had developed complications and were later shifted for cesarean delivery, the remaining 33 delivered after the observation period of the research team.

We purposively selected postnatal women to participate in face-to-face interviews in the postnatal ward. Selection criteria of the postnatal women were delivery of the women in the labor room of the study medical school within the last 24–48 hours of interview, history of uncomplicated labor and delivery, history of no postpartum complication, delivery of healthy newborn, and willingness to participate in the study. All the postnatal women who met the above criteria were enrolled in the study consecutively.

We obtained written permission and consent from the head of the department of obstetrics and gynecology of the respective medical school for observations in the labor room. Before observation, 1 of the research team members introduced themselves to the laboring women. The research team member read the participant information sheet (Supplement 1) to the women in the language they understood, including the right to refuse and withdraw consent. All pregnant women gave their verbal informed consent before observation in the labor room. The research team collected background information from the women, including age, address, living arrangement, gravida, parity, education of the women and head of the family, occupation (formal employment) of the women and head of the family as per modified Kuppuswamy scale for socioeconomic status.^25^ We obtained verbal informed consent from the health care providers in the labor room to observe practices. To ensure confidentiality and privacy of the pregnant women, only the attending doctor and 1 researcher were present during delivery in the cubicles, which were covered with curtains in the labor room. We gave all postnatal women the participant information sheet, and they gave their written informed consent before the interview. At the end of the visit, the research team debriefed the observations with the head of the department and co-investigators.

Usually, in the labor rooms of a medical school, a junior faculty member is in-charge on a rotation basis, and senior consultants are on call. Resident doctors are posted in the labor rooms on a 12-hour rotation basis. They assist in clinical rounds, fill case sheets, send blood samples to the laboratory, conduct normal deliveries, and prepare women for labor and cesarean deliveries. Nurses assist the doctor during the preparations for labor and delivery, per vaginal examination and surgical procedures in the labor room. Nurses also maintain the records, conduct labor room census, administer the medications prescribed by the doctors, and monitor the maternal and fetal vital signs. Eight of the 9 medical schools had both undergraduate and postgraduate medical teaching. In these medical schools, the junior residents who were postgraduate trainees in the obstetrics and gynecology departments assisted vaginal deliveries under the guidance of senior residents and faculty in charge. In 1 medical school with only an undergraduate teaching program, postgraduate doctors assisted all deliveries (senior residents/faculty).

#### Data Collection Tools

A paper-based checklist for observation of evidence-based intrapartum care practices (Supplement 2) and interview schedule of postnatal women (Supplement 3) was developed in consultation with a core group consisting of 12 faculty and experts from obstetrics and gynecology, and public health. They consulted and reviewed published literature including Cochrane reviews, the WHO health library, and WHO recommendations for intrapartum care for a positive childbirth experience to narrow down to the practices in each stage of labor and immediate postpartum care.^1^ Those practices that were likely to impact the mortality or morbidity, affect the childbirth experience of the women, and be easily assessable and changed with the intervention were shortlisted. This checklist was pretested in 3 sites not included in the study. The checklist included the recommended and not-recommended practices during labor as per the WHO intrapartum care guidelines for the positive childbirth experience^1^ (Table 1).

**TABLE 1.** WHO Intrapartum Care Guidelines Recommended and Not-Recommended Practices During Labor for Positive Childbirth Experience Among Low-Risk Pregnant Women

Recommended Practices	Not-Recommended Practices
First stage of labor	
Using partograph to assess the progress of labor Offering alternate/upright birthing positions to laboring women Encouraging mobility during labor Performing per vaginal examination at an interval of 4 hours	Doing routine perineal/pubic shaving Performing routine enema on admission Augmenting with intravenous oxytocin prior to confirmation of delay in labor
Second stage of labor	
Encouraging the adoption of the birthing position of choice including upright (semi-recumbent, squatting, and sitting) positions	Applying manual fundal pressure Routinely or liberally using episiotomy
Third stage of labor	
Prophylactically administering uterotonics immediately after the birth of the child	
Neonatal care	
Wrapping the baby immediately after birth Delaying cord umbilical cord clamping (not earlier than 1 minute) Providing immediate skin-to-skin contact of the newborn with the mother (newborn put on mother's abdomen) Initiating early breastfeeding within 1 hour of birth	Routinely suctioning of nose and throat of the newborn in all cases
All stages of labor	
Providing respectful maternity care throughout the labor Allowing a companion of choice throughout labor and childbirth	

Abbreviation: WHO, World Health Organization.

Source: WHO recommendations on intrapartum care for positive childbirth experience.

As per WHO recommendation on intrapartum care for a positive childbirth experience, augmentation of labor is the process of stimulating the uterus to increase the frequency, duration, and intensity of contractions after the onset of spontaneous labor. For judging unindicated augmentation, we used WHO's guiding principles for augmentation of labor, which states that “augmentation of labor should be performed only when there is a clear medical indication and the expected benefits outweigh the potential harm,” and reviewed the laboring woman's case file to judge whether augmentation of labor was indicated/unindicated. The practice of augmentation was deemed not recommended when no clear medical indication of augmentation was mentioned in the case file. Augmentation through the use of pitocin/oxytocin/misoprostol was included, but no other means of augmentation (e.g., artificial rupture of the membrane) was included in the study. As per the WHO recommendations, per vaginal (PV) examination should not be done sooner than every 4 hours during labor. Even if labor lasts for 24 hours, there should not be more than 6 PV examinations; thus, performing PV examinations 6 times or more is not recommended.^1^ The experts felt that those practices that could not be observed easily, such as assessing the frequency of uterine contraction, should not be included in the checklist. These were assessed indirectly by documenting the use of partograph and the progress of labor.

Interviews of postnatal women recorded their experience of intrapartum care, neonatal care, and respectful maternity care practices. They were not asked regarding augmentation of labor, use of partograph along with the progress of labor, and immediate neonatal care (routine suction of nose and throat of the newborn and delayed cord clamping) as these practices were essentially given by the service provider without involving her.

#### Data Analysis

The data captured in the paper-based checklist and interview schedule of the postnatal women were entered into Microsoft Excel. Data analysis was done using IBM SPSS version 22.^26^

### Qualitative Study

A constructivist grounded theory approach was used to explore faculty perceptions toward evidence-based practices.^27^ It focused on generating new theories through inductive analysis of participants' data rather than preexisting theoretical frameworks. We used the COM-B framework to provide insight into its 3 determinants, including capability, opportunity, and motivation, which influenced behavior [here, adherence to evidence-based practices] to design need-based interventions at appropriate levels to facilitate behavior change.^28^

#### Study Population

Faculty in the departments of obstetrics and gynecology in 9 medical schools in 3 states in India.

#### Sample Size and Sampling Technique

We purposively selected the faculty for conducting in-depth interviews. Selection criteria for the faculty were involvement in teaching undergraduate students, especially normal vaginal delivery and intrapartum care practices, either posted or in charge of the labor room, and willingness to provide the information through in-depth interviews. We adopted the in-depth interview method as it has the advantage of eliciting detailed information about the interviewees' behaviors, attitudes, and perceptions and provides the interviewer freedom of additional probing of the participants as necessary. Data were collected till data saturation. Five faculty from each institute were approached for the interview, of which 3–4 faculty per institute were interviewed. We conducted a total of 33 interviews.

#### Data Collection Tools

A pretested in-depth interview guide was used comprising questions related to evidence-based intrapartum care practices, respectful maternity care, logistics, and opportunity for refresher training/continued medical education and reading habits of faculty themselves (Supplement 4). We explored information on continuing professional development practices among the faculty and their opinions on existing teaching development programs and their participation in such programs or continued medical education, especially regarding intrapartum care practices.

#### Data Collection Methods

We conducted a face-to-face interview (for 50–70 minutes) with each faculty member at a place convenient to him or her. Faculty provided written informed consent for both audio recording and in-depth interviews. If they did not give consent for audio recording, we asked for permission for a handwritten recording (manual) of the interview notes on a register. In such a scenario, the project officer conducted the interview and the research associate recorded the interview notes manually. We took 4 manual and 29 audio recordings. Two researchers conducted all the faculty interviews. The senior researcher asked the questions, and the other researcher was the rapporteur. Efforts were made to mitigate personal bias. The researchers were trained to conduct interviews. As per the availability, some faculty interviews were also conducted by the faculty from the central coordinating institute. The participant information sheet and informed consent form were shared with the faculty before the interview. Strict privacy and confidentiality were ensured. The rigor of the data was ensured as the same researchers conducted all the interviews. Data were collected until saturation.

#### Data Analysis

We transcribed the audio recordings, translated them into English, and entered them into an Excel spreadsheet. The transcripts were read and reread by the 3 authors (project officer and research associate) independently and later reviewed by the third author (faculty). Thematic analysis was done manually to identify the memos, codes, subthemes, and themes and later mapped on the subcomponents of the COM-B framework using the constant comparison technique. An external researcher was consulted to critique the analysis, and the suggestions were later incorporated.

#### Ethical Approval and Permission

The Institutes' Ethics Committee approved this study at the central coordinating institute (PGI/IEC/2018/001270) and medical schools in UT and Gujarat, respectively. In Rajasthan, the DMER provided the permission, and we did not need to obtain separate ethical permission to conduct the study in the medical schools in this state. We obtained written permissions from the director and controller of all participating medical schools, as well as written permission and consent from the heads of the department of obstetrics and gynecology of the respective medical schools for observations in the labor room.

## RESULTS

### Quantitative Study

In 9 medical schools, we observed a total of 155 women (91 in Rajasthan, 45 in Gujarat, and 19 in UT) during labor and immediately after childbirth. The characteristics of the women observed in the labor room are presented in Table 2. Women's mean age was 24.5±3.9 years (range 23–27 years), more than half were illiterate, about half were primigravida, and 80% stayed in a joint family (where 2 or 3 generations live together). All the laboring women were full term with the range of 37–40 weeks (maximum 39+ gestational age) without any known complication, falling under the category of healthy pregnant women per WHO guidelines.^1^

**TABLE 2.** Characteristics of Women Observed in the Labor Room and Faculty Interviewed in the Study Medical Colleges, India

	No. (%)
Women observed in the labor room (N=155)	
Age group, years	
18–22	44 (28.4)
23–27	70 (45.2)
28–32	35 (22.6)
33–38	6 (3.9)
Mean age, years	24.5 ± 3.9
Gravida	
Primigravida	78 (50.3)
Multigravida	77 (49.7)
Family type	
Nuclear	31 (20)
Joint family^a^	124 (80)
Client's education level	
Professional/post-graduate/graduate/honors	21 (13.5)
Intermediate/post-high school diploma/high school	28 (18.0)
Middle school	13 (8.5)
Primary school or literate	11 (7.1)
Illiterate	82 (52.9)
Client occupation	
Professional/clerical/shop owner/farmer	6 (4.0)
Skilled/semi-skilled/unskilled worker	9 (5.7)
Unemployed	140 (90.3)
Faculty interviewed in-depth (N=33)	
Male	8 (24.2)
Female	25 (75.8)
Age group, years	
38–44	8 (24.2)
45–54	21 (63.6)
55–64	4 (12.1)
Total teaching experience, years	
1–11	20 (60.6)
11–21	8 (24.2)
21–31	4 (12.1)
31–41	1 (3.0)
Position	
Professor	8 (24.2)
Associate professor	14 (42.4)
Assistant professor	10 (30.3)
Senior medical officer	1 (3.0)

a Where two or three generations live together.

The intrapartum care practices of service providers toward 100 laboring women in the first, second, and third stage of labor, 35 women in the first stage only, and 20 in the second and third stage of labor were observed (Table 3). Recommended evidence-based intrapartum care practices that were observed included partograph used along with the progress of labor in 39.3% cases, presence of birth companion in 69.6% cases, fetal heart rate assessment in 100% of cases (hourly assessment in 82.2%; half-hourly assessment in 17.8%); uterotonics administered on time to 90.8% women; verbal consent obtained before PV examination from 63.7% women; curtains drawn during PV examination for 31.8% women; concern and empathy shown to 70.8% women, and 76.6% women helped to move around during labor.

**TABLE 3.** Observations of Intrapartum Evidence-Based Practices, Neonatal Care Practices, and Respectful Maternity Care in the Labor Room in the Study Medical Schools in India

	No. (%)	95% CI
Intrapartum care (N=135)		
First stage of labor		
Recommended practices		
Partograph used along with the progress of labor	53 (39.3)	30.9, 48.0
Women's mobility encouraged during labor	94 (69.6)	61.7, 77.2
Offering alternate/upright birthing positions to laboring women	0 (0)	0
Frequency of PV examination (as per records)		
0–3	64 (47.5)	38.7, 56.1
4–7	70 (51.9)	43.1, 60.5
8–11	1 (0.7)	0.0, 4.0
A birth companion was allowed	94 (69.6)	61.7, 77.2
Fetal heart rate measured	135 (100)	97.3, 100
Frequency of fetal heart rate monitoring		
Hourly	111 (82.2)	74.7, 88.2
Half-hourly	24 (17.8)	11.7, 25.2
Not-recommended practices		
Pubic shaving done	54 (40.0)	31.6, 48.7
Enema given	65 (48.1)	39.4, 56.9
Augmentation with intravenous oxytocin before confirming delay in labor	87 (64.4)	55.7, 72.4
Second stage of labor (N=120)		
Recommended practices		
Alternate birthing positions were used	16 (13.3)	7.8, 20.7
Not-recommended practices		
Fundal pressure given	61 (50.8)	41.5, 60.1
Episiotomy done	70 (58.3)	48.9, 67.3
Third stage of labor (N=120)		
Recommended practice		
Uterotonics being given on time	109 (90.8)	84.1, 95.3
Neonatal care		
Recommended practices		
Baby was wrapped immediately	58 (48.3)	39.1, 57.6
Skin-to-skin contact of the child with mother (baby kept on mother's stomach immediately after birth)	41 (34.2)	25.7, 43.3
Delayed cord clamping	53 (44.2)	35.1, 53.5
Breastfeeding initiated within one hour of delivery	18 (15.0)	9.1, 22.7
Not-recommended practices		
Suctioning of throat and nose of newborn done	41 (34.2)	25.7, 43.3
Respectful maternity care		
PV examination (N=135)		
Verbal consent was taken from the client before PV examination	86 (63.7)	54.9, 71.8
Curtains were drawn during PV examination/separate room for PV	43 (31.8)	24.1, 40.4
Client treated with concern and empathy (N=120)	85 (70.8)	62.7, 78.9
Clients helped to move around during labor	92 (76.6)	69.1, 84.2
Disrespectful maternity care		
Clients shouted at	18 (15.0)	9.1, 22.7
Clients taunted	22 (18.3)	11.8, 26.4
Clients slapped	2 (1.6)	0.2, 5.8

Abbreviations: CI, confidence interval; PV, per vaginal.

Recommended evidence-based intrapartum care practices that were observed in most women included having a birth companion, assessing fetal heart rate, and administering uterotonics on time.

Not-recommended intrapartum practices observed were pubic shaving in 40% of women, enema in 48.1% of women, and unindicated augmentation of labor (cases where no clear medical indication of augmentation based on partograph findings) in 64.4% cases. Among the women whose labor was augmented, fetal heart rate was measured hourly in 87.3% of cases and half-hourly in 12.6% of women and a partograph for the progress of labor was being used only in 39% of women (Supplement Table S1). Only 13% of women were encouraged to adopt different birthing positions during the second stage of labor, while a majority (86.7%) of women delivered in the lithotomy position. Fundal pressure was applied in 50.8% of women, and episiotomy was performed in 58.5% of cases. Among primigravida (n=78), 56.4% of women were given an episiotomy. Indicators of respectful maternity care showed that 14.4% of women were shouted at, 17.6% were taunted, and 1.6% were slapped.

Recommended neonatal care practices observed were wrapping the baby immediately for 48.3% of newborns; skin-to-skin contact of mother and baby immediately post-delivery in 34.2% of cases, delayed cord clamping in 44.2%, and breastfeeding initiated within 1 hour of delivery in 15% of cases. Suctioning of the nose and throat was practiced in 34.2% of newborns among the not-recommended practices.

We interviewed 136 postnatal women in the postnatal ward (Supplement Table S2). They reported similar findings regarding adherence to evidence-based intrapartum care practices, neonatal care practices, and respectful maternity care in the labor room (Supplement Table S3).

### Qualitative Study

We conducted in-depth interviews with 33 faculty. Nearly 63.6% were 45–54 years old, and 60.6% had up to 11 years of work experience (Table 2). The thematic analysis of the perceptions of the faculty regarding IP-EBP during the first, second, and third stages of labor is summarized in Supplement Table S4. The themes, subthemes, and codes regarding 3 crucial evidence-based intrapartum care practices, including augmentation of labor, alternate birthing position, and episiotomy, are given in Supplement Table S5.

### Faculty Perceptions Regarding Crucial Evidence-Based Intrapartum Care Practices

#### Augmentation of Labor

The majority of faculty opined that augmentation of labor is practiced to reduce labor duration to deal with the high workload in busy labor rooms in the hospitals and avoid maternal complications.

*With more preference of daytime obstetrics, we prefer that sooner the patient delivers the better it is …. in our institute almost 50% deliveries are augmented, we do not keep patient in continuous labor for too long*. —Professor, 10 years' experience, Gujarat

Faculty also expressed that labor should not be augmented routinely and done only when indicated (like when labor is not progressing), as augmentation can lead to various complications, including fetal distress, meconium-stained liquor, and increased chances of cesarean delivery. However, they also said it poses no threat if prior ultrasound is normal and close monitoring is done.

*Augmentation needs to be ascertained from case-to-case basis and should not be used in routine.* —Professor, 10 years' experience, Gujarat

#### Upright Position During Delivery

Some faculty believed that alternate birthing positions should be promoted.

*If a multigravida wants to deliver in a different position like standing or squatting, we don't deny it.* —Assistant professor, 10 years' experience, Rajasthan

The majority felt that lithotomy is practiced due to untrained labor room staff and lack of confidence to conduct delivery in other positions, overcrowding in the labor room, lack of infrastructure and space, and fear of injury to the baby.

*[We deliver most women in) supine or lithotomy position, other (positions are) practically not possible, we are not conformable or used to or trained to handle other positions.* —Associate professor, 13 years' experience, Rajasthan

#### Episiotomy

As per faculty, episiotomy should be done routinely, especially in primigravida, to prevent fetal complications, and perineal tear. They also reported that a lack of confidence in service providers to conduct normal vaginal delivery (without episiotomy) might also lead to increased hospital episiotomy rates.

*Single episiotomy cut is easier to manage in terms of suturing.* —Associate professor, 13 years' experience, Rajasthan

Some others opined that episiotomy is given only if indicated in cases of rigid perineum, good size baby, and to prevent maternal and fetal complications.

#### Other Recommended Intrapartum Practices

Factors promoting and preventing other recommended practices are given in Supplement Table S1.

Some faculty members supported that pubic shaving is not required, and an enema should not be given as it can irritate the gut. Fundal pressure should not be given, leading to complications like uterine prolapse. Having a birth companion of choice made a difference for the women.

*There were high chances of normal delivery in the presence of birth companion.* —Assistant Professor, 8 years' experience, Rajasthan

Some faculty promoted pubic shaving and enema for hygiene and cleanliness.

*Pubic shaving and enema are part and parcel of clean practices and required for episiotomy.* —Assistant professor, 10 years' experience, UT

They also said that barriers to hair clipping included having untrained staff and longer time consumed for hair clipping compared to shaving. Enema was being practiced since it helped to rule out false labor.

Fundal pressure was rarely given to manage the second stage of delivery, especially in the government setups.

*[Fundal pressure] helps in second stage of labor, it is usually practiced by other labor room staff in the absence of faculty members.* —Associate professor, 10 years' experience, Rajasthan

Birth companion of choice during labor and delivery might not be possible due to insufficient arrangements for privacy.

*[We have] no visual privacy, so male attendants cannot be allowed.* —Associate professor, 8 years' experience, UT

Privacy during labor and delivery could not be maintained due to overcrowding and lack of infrastructure.

Sometimes, exhaustion due to overwork lowers the threshold of residents and staff, and as a result, they can quickly raise their voices or behave rudely with the clients or their attendants.

*Patients and doctors are also sometimes touchy, tolerance threshold of the patients and doctors have decreased.* —Associate professor, 8 years' experience, UT

Providers conducted digital or PV examination at 4 hourly intervals for routine assessment of labor in low-risk women 4 to 7 times in 51.9% of cases and 0 to 3 times in 47.5% of cases. Providers obtained verbal consent before PV from 63.7% of women. Some faculty members believed that they did not need explicit verbal consent for conducting a vaginal examination as the woman had already given her implied consent by visiting the outpatient department/labor room for treatment.

The Figure shows barriers perceived by the faculty to adhere to IP-EBP as per the COM-B model (physical and psychological capability; social and physical opportunity; automatic and reflective motivation). We observed that faculty with more than 10 years of experience favored not doing routine augmentation and routine episiotomy unless indicated; also, they wanted to offer alternate birth positions or positions of choice for delivery to the patient. However, the faculty with less experience were more likely to follow practices as per convenience. For example, an assistant professor from Rajasthan reported that “70–80% patients were augmented due to high workload.” Only 12 faculty (36.6%) members mentioned having attended a continued medical education/workshop on reproductive rights in a year organized by state-specific obstetrics and gynecology societies.

**FIGURE:**
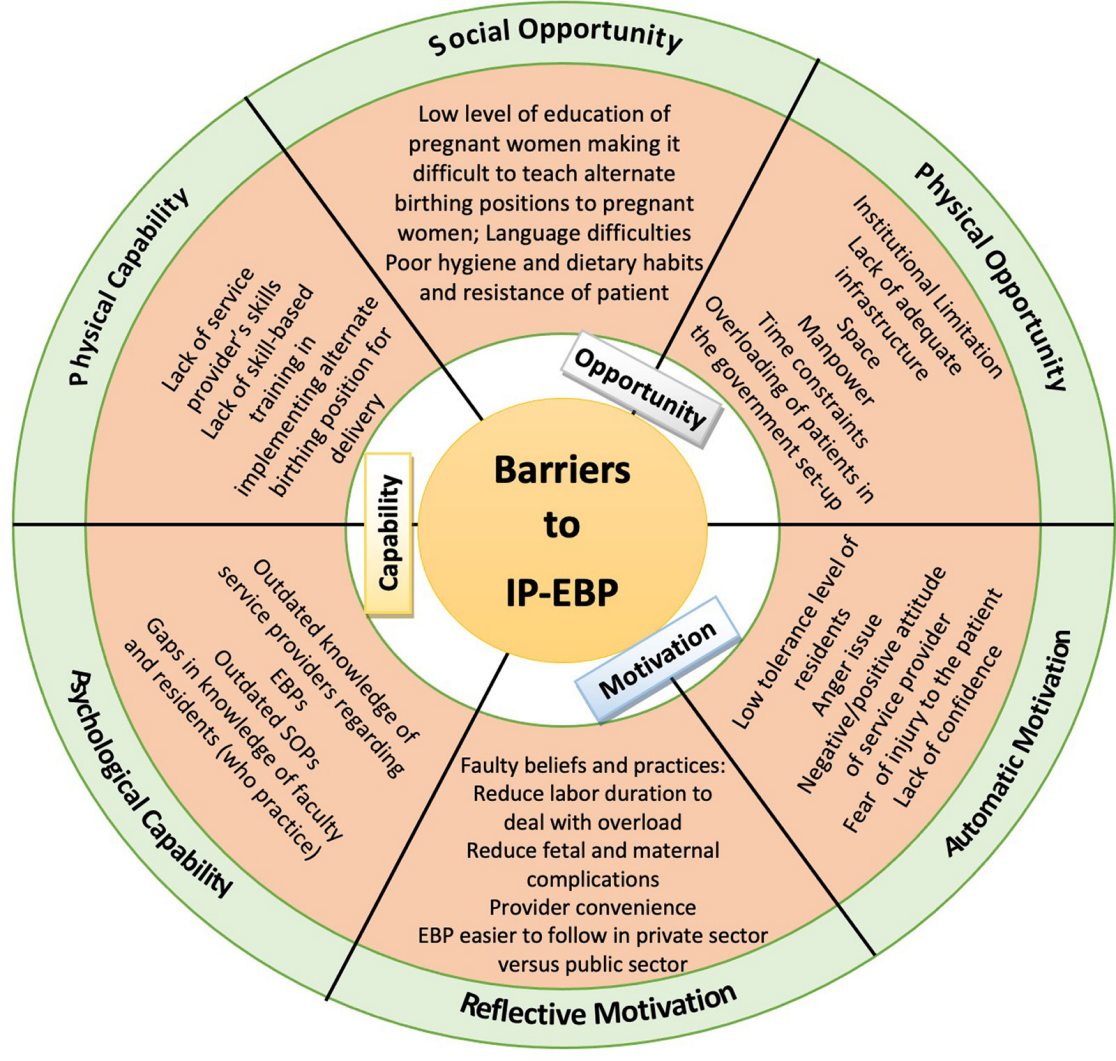
Barriers Perceived by Medical School Faculty in India to Adhere to Intrapartum Evidence-Based Practices per the Capability, Opportunity, and Motivation Behavior Model Abbreviations: EBP, evidenced-based practice; IP-EBP; intrapartum evidenced-based practices; SOP, standard operating procedure.

## DISCUSSION

We identified significant gaps between current intrapartum practices and recommended guidelines, especially for augmentation of labor, fundal pressure, routine episiotomy, and offering alternate birthing positions by the service providers in the medical schools in India. Though several studies have assessed the quality of intrapartum care practices in public health facilities, there are very few studies on intrapartum practices in medical schools.^15^^,^^29^ Hence, this study adds crucial evidence to the available public health knowledge.

In our study, some of the not-recommended practices (pubic shaving in 40%, enema on admission in 51.9%, episiotomy in 58.3%, and fundal pressure in 50.8%) were commonly practiced. Service providers believed that pubic shaving and enema were essential parts of hygienic practices and required to prevent infection and facilitate episiotomy, although there is insufficient evidence to recommend perineal shaving for women in labor as per a Cochrane review.^30^ Augmentation of labor observed in 64.4% of cases was supported by the faculty's belief that augmentation was needed to accelerate labor in busy and overcrowded labor rooms reflecting adherence to a traditional belief and an infrastructural gap. This finding is in line with a study in Rajasthan reporting routine augmentation in 93% of public health facilities.^9^ A multicountry study from Indonesia, Malaysia, Thailand, and the Philippines reported liberal use of episiotomy varying between 47 to 91%, similar to this study.^31^ More frequent vaginal examinations during labor and lack of consent have been reported in other studies from India.^8^^,^^17^ Often, the consent for PV examination was not taken as the faculty believed that the consent is implied when the patient came to the OPD/labor room for the treatment. However, the legal guidelines mandate that explicit verbal consent should be taken for conducting any intimate examination including PV examination during labor.^32^

Similar to our study, the near-universal use of lithotomy or the dorsal position was reported in other studies from India as well.^15^^,^^19^ The dorsal position is also widely used in other settings, such as Africa.^19^ The reasons cited in the African study for not using alternate birthing positions were lack of training and skills in conducting delivery in upright positions, personal comfort, and lack of equipment or facilities, which are similar to our study. Though few providers reported that they sometimes encourage women to deliver in alternate positions, observations showed that nearly all deliveries were conducted in the dorsal/lithotomy positions. Interestingly, a study from Brazil showed that alternate positions for childbirth were used in nearly one-fourth of delivery before the intervention, which increased to 40% after the intervention.^33^ Studies on knowledge of obstetric care providers (including doctors, nurse-midwives, and midwives) in Kenya also revealed substantial gaps in knowledge of providers.^34^ Fundal pressure application by the health care provider of 50.8% was supported by the erroneous belief of faculty that it helped in delivery during the second stage, which was not as per recommended practice given in WHO guidelines on fundal pressure. Low certainty in the evidence suggests that women receiving manual fundal pressure may experience more pain after birth (assessed in terms of analgesic requirements) than those not receiving fundal pressure.^1^ This practice was higher (50.8%) in this study than in a study (30.6%) in Turkey.^8^ WHO recommends an episiotomy rate of 10%,^1^ which is one-sixth of what we observed in our study (58.3%). Faculty felt that episiotomy was to be done routinely in 90% of primigravida and cited reasons. Evidence shows that episiotomy is more likely to lead to third- or fourth-degree perineal tears than first- and second-degree tears in normal vaginal delivery without episiotomy.^35^ They also reported that since postgraduate students assisted uncomplicated deliveries, the episiotomy rate was higher as students still perform routine episiotomy, reflecting habitual motivation and behavior. This finding indicated a considerable gap in the knowledge of faculty and actual practices performed by their students.

A female birth companion was present in our study with 69.6% of women throughout labor and childbirth. Evidence shows that the presence of a birth companion helps reduce the length of labor, reduce the need for intrapartum interventions (cesarean and instrumental assisted vaginal deliveries), and increase spontaneous vaginal delivery.^36^ This is supported by faculty opinions.

*Birth companion makes a hell lot of a difference for the women, helps to make her comfortable.* —Associate professor, 8 years' experience, UT

However, there are advantages and disadvantages.

*Birth companion some time interfere with the line of treatment and may compromise the privacy of other women in labor*. —Assistant professor, 1 year experience, Rajasthan

Fetal heart rate monitoring at hourly intervals and overall monitoring observed in this study were in line with the International Federation of Gynecology and Obstetrics recommendations^37^ and better than the findings at tertiary care centers in the Philippines.^38^

Observations of disrespectful maternity care were less in this study, which might be due to social desirability bias.^22^ Respectful maternity care, as seen in our study, was better as a result of reflective motivation, as compared to the findings reported by Bohren et al., where mistreatment was 40%.^24^ Gaps observed in adherence to essential newborn care practices come down significantly when early essential newborn care is implemented.^21^

In exploring the reasons for nonadherence to recommended evidence-based practices as per the COM-B model, our study showed multiple barriers, such as lack of space, resources, time, as well as outdated faculty knowledge and beliefs. For example, augmentation was perceived to be necessary to accelerate labor in busy and overcrowded labor rooms. Using cues from the COM-B framework, we summarized the actionable points in Table 4 to increase the adherence to IP-EBP. Some studies have shown that continuous quality improvement initiatives using education, performance feedback, and the Hawthorne effect have resulted in a reduction in the episiotomy rate in large academic institutions.^26^^,^^27^

**TABLE 4.** Problem-Based Approach and Actions Required to Improve Adherence to Evidence-Based Intrapartum Practices in Labor Rooms

Level	Problem	Actions Required
Individual (service provider)	Attitude of the service provider Perceptions regarding time constraint Perceptions regarding increased workload in the labor room Traditional beliefs and intrapartum practices Lack of knowledge regarding the latest evidence-based practices Lack of training and technical difficulty in practicing evidence-based practices Poor skills of the junior service provider	Upgradation of knowledge and skills of the service providers regarding evidence-based practices through continuous medical education Teaching and training of junior doctors in recommended practices Imparting skills on effective time management Reinforce existing good practices by monitoring, supervision, and providing incentives Advocacy with medical councils regarding revised curriculum to include evidence-based practices and related provision of infrastructure Advocacy with the textbook writers to include evidence-based practices
Institute	Lack of sufficient number of beds Lack of space Inadequate human resources	Minor labor room modifications to allow non-supine positions Train existing manpower on evidence-based practices
Community	The prevailing perception of the laboring women regarding intrapartum practices	Sensitization of women during antenatal period regarding recommended evidence-based intranatal practices

Our study showed multiple barriers to adherence to evidence-based practices, such as fear of injury; lack of space, resources, and time; and outdated faculty knowledge and beliefs.

A key strength of this study was the use of the mixed-methods study design, a standardized pretested checklist for observation in the labor room, and an in-depth interview guide. The similarities in the quantitative and qualitative results provided evidence for the validity of the quantitative findings. The direct observation method was used to present the actual adherence status to evidence-based practices in the labor room, and it has been shown to better capture practices than interview methods.^39^ A constructivist grounded theory approach was used to explore faculty perceptions toward evidence-based practices. It focused on generating new theories through inductive analysis of the data gathered from participants rather than from preexisting theoretical frameworks. We used the in-depth interview method for interviewing faculty to elicit details about the inter-viewees' behaviors, attitudes, and perceptions and provide freedom of additional probing to the interviewer and participants as necessary.^39^ Quantitative studies documented the actual practices observed, and qualitative studies attempted to capture the perception and beliefs about IP-EBP contributing to the practices.^40^

Implications of our study's findings are that the tertiary care medical schools should serve as role models for service providers working at primary and secondary health care facilities. Furthermore, the effects of in-service training programs on doctors and nurses revealed that providers are less likely to change their practices if they are contrary to what they learned in medical schools. We recommend that measures are taken to improve the adherence to evidence-based intrapartum care practices in medical schools, which is expected to improve the quality of teaching provided to medical students and provide guidance to the more extensive public health system. Interventions to improve maternal health care services are needed, as in states like Bihar in India, where neonatal mortality is still relatively high and utilization of maternal care services is poor.^41^ Early use of intravenous oxytocin for slow progress in the first stage of labor leads to uterine hyperstimulation with fetal heart rate changes and severe neonatal morbidity and perinatal mortality.^42^ Also, promoting alternate birthing positions (upright) decreases instrument-assisted deliveries, episiotomy, and cesarean deliveries and is associated with fewer abnormal fetal heart rate patterns.^43^ But, to support a woman giving birth in a recumbent/alternate position, the health care providers require training and adequate infrastructure. The policy of routine use of episiotomy may act as a significant barrier to uptake of facility-based birth by disadvantaged women in low- and middle-income countries "due to fear of cutting" (cesarean delivery and episiotomy).^44^ Therefore, placing a clearly communicated policy of selective/restrictive episiotomy with emphasis on informed consent for the invasive procedure will help governments to increase facility-based birth coverage among disadvantaged women.^45^

### Limitations

The study findings are limited in generalizability as sampling was not done at the level of medical schools, medical schools were not selected randomly in the states, and the women were observed consecutively on the visit to the labor room. Facility-specific sample size was not estimated due to operational and feasibility issues to conduct the study. Conducting the study as per institution-wise sampling might have increased the study's generalizability to the specific institute, but it would not have been economically viable due to limited funds availability. Also, the study had to be completed within a stipulated timeframe. The time of onset of labor and the duration between admission to the labor ward and birth of the baby was not documented; hence, it might be challenging to ascertain the actual risk status of women regarding the progress of labor. However, we reviewed the case files to find out the underlying medical reasons for complications during labor that could have led to augmentation of labor or high-risk status of women. To minimize the Hawthorne effect, service providers in the labor room were told that data collection was anonymous and individual performance would not be reported. The providers were briefed about the overall observation procedure as given in the participant information sheet without sharing the specific details of the checklist's parameters. In addition, postnatal women were also interviewed to support the observational findings (Supplement Table S2). Evidence shows that the the Hawthorne effect diminishes over time; the longer the observation period, the lesser the effect will be.^46^ Since the investigators did not mention the practices to be measured to the service providers, this reduced the social desirability bias. The potential observer's bias in data collection and interpretation of results was mitigated using a standardized checklist. Detailed facility-wise infrastructure and resource assessment was not done, as that was not the study's objective. Challenges were mainly faced during qualitative data collection in terms of faculty availability due to their busy schedule.

## CONCLUSION

To substantially improve maternal and neonatal outcomes, the quality of maternal and neonatal care needs to be improved by training health care providers to provide skilled care, as well as ensuring they have adequate supplies and infrastructure.^47^ Our study is significant as it provides us with the basis to design targeted interventions and achieve significant quality-of-care and cost-effective benefits. Further, improved practices among medical educators are expected to enable better support to the midwifery cadre, scale up evidence-based birthing practices, and improve maternal and perinatal outcomes. For this to be achieved, efforts are needed to advocate with medical councils and textbook writers regarding updating textbooks, build the capacity of medical educators and staff, and provide resources to support changes in practice, for example, minor labor room modifications to allow nonsupine positions for delivery. At the community level, pregnant women should be sensitized during the antenatal period regarding recommended practices in the labor room (Table 4). A systemic review on the effectiveness of quality improvement collaborative strategies in low- and middle-income settings showed that quality improvement collaboratives, when combined with other interventions like training health staff, are more effective in improving the quality.^48^ Intervention research needs to be conducted to shed light on optimum and need-based interventions to bring about these changes in medical school practices in the future.

## Supplementary Material

21-00590-Gupta-Supplements-1-4.pdf

21-00590-Gupta-Supplement-Tables-1-5.pdf

## Notes

**Peer Reviewed**
