# Supplementary material for: Baseline Assessment of Evidence-Based Intrapartum Care Practices in Medical Schools in 3 States in India: A Mixed-Methods Study
Source: Glob Health Sci Pract. 2022 Apr 28;10(2):e2100590. doi: 10.9745/GHSP-D-21-00590 (PMC9053154; doi:10.9745/GHSP-D-21-00590)
Supplement: 21-00590-Gupta-Supplements-1-4.pdf [file 21-00590-Gupta-Supplements-1-4.pdf]

Supplement to: Iyengar K, Gupta M, Pal S, et al. Baseline assessment of evidence-based intrapartum care practices in medical schools in 3 states in India: a mixed-methods study. *Glob Health Sci Pract*. 2002;10(2):e2100590. https://doi.org/10.9745/GHSP-D-21-00590

SUPPLEMENT 1. PARTICIPANT INFORMATION SHEET (PIS) AND INFORMED CONSENT FORM (ICF)

PROTOCOL NO:
SPONSOR: UNFPA New Delhi

PRINCIPAL INVESTIGATOR:
Dr. Madhu Gupta, Professor, Department of Community Medicine, School of Public Health, Post Graduate Institute of Medical Education and Research Chandigarh, India.

Name of Participant:

Title: **Strengthening Evidence-based Family Planning Service in Pre-service Medical Education**

This project is planned with the objective to improve the quality of care and adherence of evidence-based, especially evidence based maternal care practices, reproductive rights, medical eligibility criteria of contraceptives etc, standard clinical practices in medical colleges, focusing on maternal care and family planning and to improve the quality of teaching provided to medical students, so that it builds greater skills for family planning and respectful maternity care and sensitivity towards reproductive rights.

1. You are invited to take part in this research study. The information in this document is meant to help you decide whether or not to take part. Please feel free to ask if you have any queries or concerns.

2. **What is your expected duration of the participation?**
 Your participation in this study will last for a total of one hour approximately.

3. **What procedures will be followed during this study?**
 If you agree to participate in this study, you will be asked to sign the given consent form. You will be assessed based on one-to-one observations and strict attention will be paid to confidentiality during that. This whole procedure will take approximately one hour. Thereafter, you will not be asked for any other things.

4. **What are the risks and discomforts to you?**
 There is no risk concerned as this is observational study.

5. **What benefits are expected from this research?**
 For the clients: If you participate in this study, it will help you in improving the overall family planning implementation strategies and may also help you to improve the health indicators of your area.

6. **What are the alternatives available to you?**
 This is an observational study.

7. **Are the data/records of the participant kept confidential?**
 Information's collected for this study will be kept confidential. Your records to the extent of the applicable laws and regulations will not be made publicly available. Only your Investigators will have access to the confidential information being collected.

8. **What will be the treatment schedule(s)?**
 No treatment is being given to the participants, as this is an observational study.

9. **What compensation and/or treatment(s) are available to the Participant in the event of a trial-related injury?**
 If you take part in the study, you will not receive any compensation for participating in this study.

10. **Whom to contact for trial related queries and what are the rights of Participants in the event of any injury?**
 Your participation in this study is entirely voluntary. Your questions will be answered clearly and to your satisfaction
 `

11. **Are the participants paid to take part in this study?**
 The participants are not being paid for participation.

12. **What are your responsibilities during participation in the study?**

 If you agree to participate in this study, you just have to help us in filling the study proforma with accurate

information you will be asked for.

13. Participation is voluntary, you can withdraw from the study at any time and that refusal to participate will not involve any penalty or loss of benefits to which the Participant is otherwise entitled.

14. You or your representative will be notified in a timely manner if significant new findings develop during the course of the research which may affect your willingness to continue participation will be provided.

15. Approximate 154 participants will be enrolled in the study

Contact persons:
For further information / questions, you can contact us at the following address:

Principal Investigator:

Dr. Madhu Gupta, Professor, Department of Community Medicine, School of Public Health, Post Graduate Institute of Medical Education and Research Chandigarh, India.

Co-Principal-Investigator

Dr Vanita Suri ,Department of Obstetrics and Gynaecology, PGIMER, Chandigarh

Contact person(s):
Dr. Madhu Gupta (Principal Investigator),
Professor, Department of Community Medicine, School of Public Health, Post Graduate Institute of Medical Education and Research Chandigarh, India.
Contact No : *07087008223, 7009769629*

In case of conflicts, you can contact the Convener of our institutional ethics committee at the following address:
Dr Samir Malhotra
Department of Pharmacology
Convener, Institutional Ethics Committee
PGIMER, Chandigarh
Contact No: 0172-2755243,7087009465

Supplement to: Iyengar K, Gupta M, Pal S, et al. Baseline assessment of evidence-based intrapartum care practices in medical schools in 3 states in India: a mixed-methods study. *Glob Health Sci Pract*. 2002;10(2):e2100590. https://doi.org/10.9745/GHSP-D-21-00590

INFORMED CONSENT FORM

Title of Project – Strengthening Evidence-based Family Planning Service in Pre-service Medical Education

I agree to participate in a research project led by Dr. Madhu Gupta from the PGIMER, Chandigarh, India. The purpose of this document is to specify the terms of my participation in the project through being interviewed.

1. I have been given sufficient information about this research project through participant information sheet. The purpose of my participation as an interviewee in this project has been explained to me and is clear. My participation as an interviewee in this project is voluntary. There is no explicit or implicit coercion whatsoever to participate.

2. Participation involves being interviewed and observed by researcher from PGIMER, Chandigarh. The interview and observation will last approximately [30-40] minutes. I allow the researcher to take written notes during the interview. I also may allow the recording (by audio tape) of the interview. It is clear to me that in case I do not want the interview to be taped I am at any point of time fully entitled to withdraw from participation.

3. I have the right not to answer any of the questions. If I feel uncomfortable in any way during the interview session, I have the right to withdraw from the interview.

4. I understand that the researcher will not identify me by name in any reports using information obtained from this interview, and that my confidentiality as a participant in this study will remain secure. Subsequent uses of records and data will be subject to standard data use policies which protect the anonymity of individuals and institutions.

5. I have been given the guarantee that this research project has been reviewed and approved by the PGIMER Ethics Committee. For research problems or any other question regarding the research project, the PGIMER Ethics Committee may be contacted through Dr Samir Malhotra, Convener, Institute Ethics Committee, PGIMER, Chandigarh; 7087009465

6. I have read and understood the points and statements of this form. I have had all my questions answered to my satisfaction, and I voluntarily agree to participate in this study.

7. I have been given a copy of this consent form co-signed by the interviewer.

Participants Signature Date

________________________________ ___________________________

Researcher's Signature

________________________________ ___________________________

Supplement to: Iyengar K, Gupta M, Pal S, et al. Baseline assessment of evidence-based intrapartum care practices in medical schools in 3 states in India: a mixed-methods study. *Glob Health Sci Pract*. 2002;10(2):e2100590. https://doi.org/10.9745/GHSP-D-21-00590

SUPPLEMENT 2. CHECKLIST FOR OBSERVATION OF DELIVERY IN THE LABOUR ROOM

Unique ID										

Background Details:

1. Facility name:
2. Name of observer :
3. Date and Time of observation:
4. No of hour observed:

Part A: First Stage of Labour

1. Name of woman	2.Age of woman
3. Address of woman	4. Education of woman<br>(1: Professional or Honors,2: Graduate or Post-Graduate, 3: Intermediate or Post-High-School Diploma, 4: High School, 5: Middle School, 6: Primary School or Literate,7: Illiterate)<br>4.1 Education of head of the family<br>(1: Professional or Honors,2: Graduate or Post-Graduate, 3: Intermediate or Post-High-School Diploma, 4: High School, 5: Middle School, 6: Primary School or Literate,7: Illiterate
5. Occupation of woman<br>(1: Professional, 2: Semi-Professional, 3: Clerical, Shop-owner, Farmer, 4: Skilled worker, 5: Semi-skilled worker, 6: Unskilled worker,7: Unemployed)<br>5.1 Occupation of head of the family<br>(1: Professional, 2: Semi-Professional, 3: Clerical, Shop-owner, Farmer, 4: Skilled worker, 5: Semi-skilled worker,6: Unskilled worker,7: Unemployed)	6. Gravida: Birth order of current pregnancy (including any abortions, miscarriages or stillbirths)<br>Parity :<br>P_G_L_A

Parameter	Yes/ No	Remarks
First stage of Labour:<br>1. Is BP measured?	Yes= 1 No= 0	
2. Is fetal heart rate measure?	Y/N	
3. How frequently?	1Hourly 2. half hourly	
4. Are women being restricted to move during labour.	Yes= 1 No= 0	
5. Is birthing companion present during labour	Yes= 1 No= 0	
6. Is enema given before labour	Yes= 1 No= 0	
7. Is Partograph being used for the delivery	1Not et all<br>2.Along with progress<br>3.After labour	
8. Is pubic shaving done	Yes= 1 No= 0	
9. Was oxcytocin / miso/ serviprime given to augment the labour ?	Yes= 1 No= 0	
10. Was verbal consent taken before doing PV<br>11. No of times PV was done for each patient<br>12. Are PV findings being recorded in case files?<br>13. Were fresh gloves used for each patient?	Yes= 1 No= 0<br>Number . ___<br>Yes= 1 No= 0<br>Yes= 1 No= 0	
14. Are Curtains drawn during PV?	Yes= 1 No= 0	

Part B: Second and Third Stage of Labour

21. Name of woman	22.Age of woman
23. Address of woman	24. Education of woman<br>(1: Professional or Honors,2: Graduate or Post-Graduate, 3: Intermediate or Post-High-School Diploma, 4: High School, 5: Middle School, 6: Primary School or Literate,7: Illiterate<br>24.1**Education of head of the family**<br>(1: Professional or Honors,2: Graduate or Post-Graduate, 3: Intermediate or Post-High-School Diploma, 4: High School, 5: Middle School, 6: Primary School or Literate,7: Illiterate
25. Occupation of woman<br>(1: Professional, 2: Semi-Professional, 3: Clerical, Shop-owner, Farmer, 4: Skilled worker, 5: Semi-skilled worker, 6: Unskilled worker,7: Unemployed)<br>25.1 **Occupation of head of the family**<br>(1: Professional, 2: Semi-Professional, 3: Clerical, Shop-owner, Farmer, 4: Skilled worker, 5: Semi-skilled worker,6: Unskilled worker,7: Unemployed)	26. Gravida: Birth order of current pregnancy (including any abortions, miscarriages or stillbirths)<br>Parity :<br>P_G_L_A

Second stage of Labour<br>27.What position of bearing down in being used?	1. Squatting<br>2. Sitting<br>3. Semi recumbent<br>4. Lithotomy	
28. Is episiotomy being given	Yes= 1 No= 0	
29. Is fundal pressure being given?	Yes= 1 No= 0	
30. What is the intensity	1.Mild<br>2.Moderate<br>3.Sever	
31. How is fundal pressure being given? Specify.		
Third stage of labour :<br>32. Are Uterotonics being given?	Immediately after birth and before delivery of placenta<br>Yes= 1 No= 0	
33. Is the baby wrapped immediately?	Yes= 1 No= 0	
34. Is cord clamping delayed by 1-2 mins?	Yes= 1 No= 0	
35. Is the baby kept on mother's stomach immediately after birth?	Yes= 1 No= 0	
36. Is breastfeeding initiated within one hour of delivery?	Yes= 1 No= 0	
37. Is suctioning of new born done?	Yes= 1 No= 0	
38. Was PPIUCD inserted?	Yes= 1 No= 0	
39. If yes, Was consent taken	1.In labour<br>2.After birth of baby	
40. Was she informed before insertion	Yes= 1 No= 0	
41. The women were being slapped the during delivery for different reasons	Yes= 1 No= 0	
42. The women were shown concern and empathy	Yes= 1 No= 0	
43. The women were being shouted at?	Yes= 1 No= 0	
44. Was the women being taunted ?		
45. Was she helped to move around during labour?	Yes= 1 No= 0	

Part C: Record Review: (Data of previous month from labour room)

46. Total deliveries:	
47. Normal vaginal delivery	
48. Instrumental assisted delivery	
49. C section:	
50. Referred out cases:	
51. Live births:	
52. Still Births	
53. No of PPIUCD inserted after C section	
54. No of PPIUCD inserted after vaginal delivery	

Part D: Information Education and Communication Material displayed and status of privacy in the Labour room

Parameter	Yes/No	Remarks
Displays:	Yes= 1 No= 0	
55. New born resuscitation poster	Yes= 1 No= 0	
56. PPIUCD eligibility checklist	Yes= 1 No= 0	
57. Cleanliness checklist	Yes= 1 No= 0	
58. Safe childbirth checklist	Yes= 1 No= 0	
59. Screen/Partitions between two tables in labour room	Yes= 1 No= 0	
60. Any other observation		

Signature of the investigator/Researcher: Name of the investigator/Researcher:

Supplement to: Iyengar K, Gupta M, Pal S, et al. Baseline assessment of evidence-based intrapartum care practices in medical schools in 3 states in India: a mixed-methods study. *Glob Health Sci Pract*. 2002;10(2):e2100590. https://doi.org/10.9745/GHSP-D-21-00590

SUPPLEMENT 3. POSTNATAL WOMAN INTERVIEW SCHEDULE

Section I: Socio Demographic Variable

1.Facility Details	2.Name
3.Age	4.Address<br>5.Phone number
6.Gravida: Birth order of current pregnancy (including any abortions, miscarriages or stillbirths)	7.No. of living children
8. Education of client<br>(1: Professional or Honors,2: Graduate or Post-Graduate, 3: Intermediate or Post-High-School Diploma, 4: High School, 5: Middle School, 6: Primary School or Literate,7: Illiterate	9.Occupation of client<br>(1: Professional, 2: Semi-Professional, 3: Clerical, Shop-owner, Farmer, 4: Skilled worker, 5: Semi-skilled worker,6: Unskilled worker,7: Unemployed)
8.1 Education of head of the family<br>(1: Professional or Honors,2: Graduate or Post-Graduate, 3: Intermediate or Post-High-School Diploma, 4: High School, 5: Middle School, 6: Primary School or Literate,7: Illiterate	9.1. Occupation of head of the family<br>(1: Professional, 2: Semi-Professional, 3: Clerical, Shop-owner, Farmer, 4: Skilled worker, 5: Semi-skilled worker,6: Unskilled worker,7: Unemployed)
10 Number of family members	11. Family type: 1. Nuclear 2. Joint
12. Family's combined income<br>1. > 126360 rs 2. 63182-126356 rs 3. 47266-63178 rs. 4. 31591-47262 rs 5. 18953-31589 6. 6327-18949 7. <=6323rs	13.(Socio-economic status as per modified Kuppuswamy 2018)

Section II: Interview schedule

14. Are you satisfied with maternal health related services provided to you?
क्या आप मातृ स्वास्थ्य से जुड़ी सेवाओं से संतुष्ट हैं?

Very much satisfied<br>1<br>बहुत संतुष्ट हैं	Satisfied<br>2<br>संतुष्ट हैं	Neither satisfied nor dissatisfied<br>3<br>न तो संतुष्ट न ही असंतुष्ट	Dissatisfied<br>4<br>असंतुष्ट	Very much Dissatisfied<br>5<br>बहुत असंतुष्ट

Whether the following was done:

Task	Yes/No	Remarks
15. Did somebody came to check you during labour?<br>If yes what was the frequency<br>क्या कोई प्रसव पीड़ा के दौरान आपकी जांच करने आया था?	Yes= 1 No= 0	
16. Did somebody measure fetal heart rate?<br>क्या किसी ने भ्रूण की हृदय गति मापी/आपके पेट पे डॉक्टर ने आला या कोई मशीन लगा कर देखा था ?	Yes= 1 No= 0	
17. Were you allowed to move during labour in the labour room?<br>क्या आपको लेबर रूम में प्रसव के दौरान घूमने फिरने की अनुमति थी/घूम फिर सकते हो ऐसा बोलै था किसी डॉक्टर ने ?	Yes= 1 No= 0	
Birth companion<br>18. Was a birth companion present with you<br>एक जन्म साथी की अनुमति थी	Yes= 1 No= 0	
19. If yes,	1.During admission/labour<br>2. At the time of delivery<br>3.Throughout Delivery/ Labour<br>यदि हाँ, तो कब:<br>1. प्रसव पीरा के दौरान<br>2.प्रसव के दौरान<br>3. पूर्ण प्रसव के दौरान	
20. If no did you needed a birth companion<br>यदि नहीं क्या आपको जन्म साथी की आवश्यकता थी	Yes= 1 No= 0	
21. Per vaginal examination was done<br>क्या निचे से जांच की क गई थी	If yes: total how many times?	
22. Was consent taken for PV<br>क्या जाँच करने से पहले अनुमति ली गई थी	Yes= 1 No= 0	

Supplement to: Iyengar K, Gupta M, Pal S, et al. Baseline assessment of evidence-based intrapartum care practices in medical schools in 3 states in India: a mixed-methods study. *Glob Health Sci Pract*. 2002;10(2):e2100590. https://doi.org/10.9745/GHSP-D-21-00590

23. Birth position (In which position did you deliver? आपने बाचा कौनसी अवस्था में पैदा किया	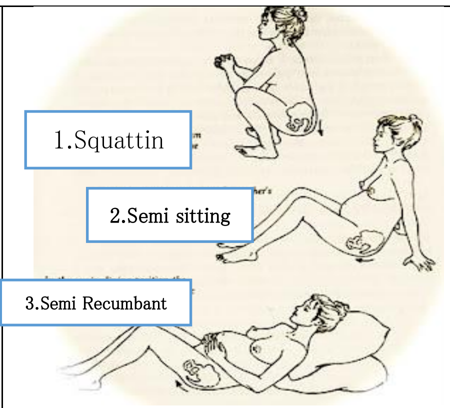	4. lithotomy<br>Lithotomy position<br>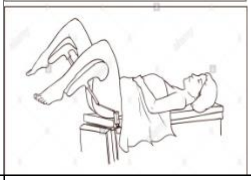
24. Were your private parts shaved? क्या आपके निचे के बाल हटाए गए?	Yes= 1 No= 0	
25. Was enema given to you? क्या आपको एनीमा दिया गया/क्या आपको लैट्रिन वाली जगह पर कोई दवाई राखी थी दर्दो के पहले	Yes= 1 No= 0	
26. Was fundal pressure given? क्या प्रसव के दौरान आपके पेट पर निचे की तरफ दबाव दिया गया?	1.Very strong;<br>2. High<br>3. Soft<br>4. Nil.	
27. Was episiotomy given to you? क्या प्रसव के दौरान आपको चीरा दिया गया/ क्या आपको नीचे ताके लगे हैं?	Yes= 1 No= 0	
28. Did you see the baby just after delivery? क्या आपने प्रसव के तुरंत बाद बच्चे को देखा?	Yes= 1 No= 0	
29. Was the baby kept on your abdomen just after delivery for few minutes? क्या प्रसव के बाद बच्चे को आपके पेट पर रखा गया?	Yes= 1 No= 0	
Breast feeding<br>30. Did you breast feed your baby within one hour of delivery? क्या आपने प्रसव के एक घंटे के भीतर अपने बच्चे को स्तनपान कराया था	Yes= 1 No= 0	
Respectful Maternity care		
31. Did anyone shout at you? क्या कोई आप पर चिल्लाया था?	Yes= 1 No= 0	
32. Did anyone taunt you? क्या किसी ने आपको ताना मारा?	Yes= 1 No= 0	
33. During labour did anyone slap you? प्रसव के दौरान किसी ने आपको थप्पड़ मारा था?	Yes= 1 No= 0	
34. Did they treat you differently due to your ethnic/caste origin? क्या उन्होंने आपके जातीय / जातिगत मूल के कारण आपसे अलग व्यवहार किया है?	Yes= 1 No= 0	
35. Did the doctor/nurse attend to you after delivery? क्या डिलीवरी के बाद डॉक्टर / नर्स आपके पास आए थे?	Yes= 1 No= 0	
Contraceptives (गर्भनिरोधक)		
36. Was PPIUCD inserted? क्या PPIUCD डाला गया था?	Yes= 1 No= 0	
37. Did they ask you before inserting PPIUCD? क्या उन्होंने PPIUCD डालने से पहले आपसे पूछा था?	Yes= 1 No= 0	
38. Did you say yes? क्या तुमने हाँ कहा?	Yes= 1 No= 0	
39. If you said no, then whose consent was taken? अगर आपने कहा कि नहीं, तो किसकी सहमति से लिया गया था?	1.पति 2.सास 3.अन्य	
40. Was any other postpartum contraceptive suggested to you क्या कोई अन्य प्रसवोत्तर गर्भनिरोधक आपको सुझाया गया था	Yes= 1 No= 0	

Record Review

41. Was episiotomy given (एपीसीओटॉमी दी गई थी?)	Yes = 1 No=0	

Signature of the investigator/Researcher: Name of the investigator/Researcher:

PIS version no. PIS dated

Supplement to: Iyengar K, Gupta M, Pal S, et al. Baseline assessment of evidence-based intrapartum care practices in medical schools in 3 states in India: a mixed-methods study. *Glob Health Sci Pract*. 2002;10(2):e2100590. https://doi.org/10.9745/GHSP-D-21-00590

SUPPLEMENT 4. IN-DEPTH INTERVIEW GUIDE FOR FACULTY

Section I: Faculty Details

1. **Name:**
2. **Age:**
3. **Designation:**
4. **Department:**
5. **College/State:**
6. **Experience in years (teaching) :**

Section II: Interview schedule:

Evidence based practices:

7. What is your perception towards pubic shaving before vaginal birth?
   i. Do you think it should be mandatory? (Yes/ No)
   ii. If yes why?
8. What birthing positions do you recommend to healthy pregnant women?
   iii. Lithotomy
   iv. Semi sitting
   v. Squatting
   vi. Other
9. Do you think that giving fundal pressure helps in delivery? (Yes/No)
   vii. Do you recommend it personally? (if yes why **)**
10. In your view what percent of women need augmentation of labor? Do you think it possess any kind of threat to the mother or the child? (if yes why**)**
11. Do you recommend routine episiotomy for primigravida ? (Yes/No)
    (if yes why **)**
12. Do you recommend enema for women having labor pains? (Yes/ No)
    (if yes why **)**
13. Do you allow birthing companion in your labor room? (Yes/ No)
    (if yes why **)**

Respectful maternity care related :

14. Are you able to maintain privacy of a client in the OPD/ Labour room? (Yes/ No)
    Use probe as needed:

    Do you face any barriers in maintaining the same in your current setting? Please let us know in brief

15. What is your perception towards the respectful maternity care of the female clients?
    a. In labor room
    b. In the OPD
    c. In the FP clinic
    Use probe as needed:

    a. Dignified care
    b. Consented care
    c. Confidential care
    d. Non abandonment in care
    e. No Physical abuse
    f. No abuse related to Cost including detention
    g. Equity in access

16. How do you ensure that the clients are not taunted, scolded or slapped during labour/delivery in your setting?
17. Do you face any challenges in incorporating and ensuring the reproductive right in the current setting?
18. Please let us know the procedure followed for PPIUCD insertion:?
    Use probe as needed:

    d. Informed consent taken- written/ informed?
19. How do you ensure that proper consent is taken by interns before conducting PV and PPIUCD insertion?

Teaching of the faculty:

20. What is your opinion on Faculty teaching development programme?
21. Does such programme exist in your medical college? (Yes/No)
22. How many CME's attended or conducted related to intrapartum care practices, family planning and reproductive health?
23. How do you think can we incorporate text book teachings on family planning in our practical teaching? Are we doing it, if not what can be the reasons for not able to do it? If yes, how do you ensure its practical teaching?
24. What Gaps do you think exists in the evidence based teaching in classroom and clinics?
25. What Suggestions would you give to strengthen the evidence based practices in clinic setting and teaching in classroom and clinics?
