# Supplementary material for: Baseline Assessment of Evidence-Based Intrapartum Care Practices in Medical Schools in 3 States in India: A Mixed-Methods Study
Source: Glob Health Sci Pract. 2022 Apr 28;10(2):e2100590. doi: 10.9745/GHSP-D-21-00590 (PMC9053154; doi:10.9745/GHSP-D-21-00590)
Supplement: 21-00590-Gupta-Supplement-Tables-1-5.pdf [file 21-00590-Gupta-Supplement-Tables-1-5.pdf]

Supplement to: Iyengar K, Gupta M, Pal S, et al. Baseline assessment evidence-based intrapartum care practices in medical schools in 3 states in India: a mixed-methods study. *Glob Health Sci Pract*. 2002;10(2):e2100590. https://doi.org/10.9745/GHSP-D-21-00590

Supplement Table S1. Frequency of fetal heart rate assessment and uterine contraction monitoring among women with routine augmentation of labour.

**Parameter**	**Number**	**%**	**95% CI**
**Number of women with routine augmentation of labour**	87	100	
**Frequency of fetal heart rate assessment in women with routine augmentation of labour**			
Hourly	76	87.3	78.5 – 93.5
Half hourly	11	12.6	6.4- 21.5
**Partograph use along with progress of labour in women with routine augmentation of labour**	34	39.0	28.7 – 50.1

Supplement Table S2. Background information of postnatal women interviewed with in 48 hours of delivery in the postnatal ward in the study medical schools.

**Number of women interviewed in postnatal ward**	**N = 136**	**%**
**Age group**		
18-22	59	43.4
23-27	56	41.2
28-32	17	12.5
33-38	4	2.9
**Gravida**		
Primigravida	64	47.0
Secondary & above	72	53.0
**Socioeconomic status**		
Upper(I)	1	0.7
Upper middle (II)	13	9.6
Lower middle (III)	57	41.9
Upper Lower (IV)	53	39.0
Lower (V)	12	8.8
**Family Type**		
Nuclear	31	22.8
Joint*	105	77.2

**Joint family where two or three generations live together*

Supplement to: Iyengar K, Gupta M, Pal S, et al. Baseline assessment evidence-based intrapartum care practices in medical schools in 3 states in India: a mixed-methods study. *Glob Health Sci Pract*. 2002;10(2):e2100590. https://doi.org/10.9745/GHSP-D-21-00590

Supplement Table S3. Status of evidence based intrapartum care, new-born care and respectful maternity care practices as perceived by postnatal women interviewed with in 48 hours of delivery in the postnatal ward in the study medical schools.

**Number of women interviewed in postnatal ward**	**N=136**	**%**	**95% CI**
**Frequency of PV examination**			
0-3	87	64.0	**55.3- 72.0**
4-7	43	31.6	**23.9- 40.1**
8-11	6	4.4	**1.6 – 9.3**
**First stage of labour**			
**Recommended practices**			
Birth companion was allowed in the labour room	91	66.9	58.3 – 74.7
**Not recommended practices**			
Pubic shaving was given before labour	58	42.6	34.2 – 51.4
Enema was given before labour	67	49.3	40.5 – 57.9
Women had restricted movement during labour.	33	24.3	17.3- 32.3
**Second stage of labour**			
**Recommended practices**			
Different birthing position were encouraged	20	14.7	9.2 – 21.7
**Not recommended practices**			
Fundal pressure was given	79	58.1	49.3- 66.4
Episiotomy was given	85	62.5	53.7 – 70.6
**New born care**			
**Recommended practices**			
Skin to skin contact of the child with mother (baby kept on mother's stomach immediately after birth) was done	44	32.4	24.5 – 40.9
Breastfeeding initiated within one hour of delivery.	33	24.3	17.3 – 32.3
**Respectful Maternity Care**			
Verbal consent was taken from patient before doing PV	106	77.9	**70.3 – 84.6**
Clients who were attended during labour	131	96.3	**91.6 – 98.8**
Clients helped to move around during labour/ attended after delivery	111	81.6	**74.0- 87.4**
Clients who were attended after delivery	111	81.6	**74.0- 87.4**
Clients who saw the baby just after delivery	111	81.6	**74.0- 87.4**
**Disrespectful practices**			
Clients Shouted at	7	5.2	2.0 – 10.3
Clients Taunted	4	2.9	0.8- 7.3
Clients Slapped	4	2.9	0.8- 7.3
**Perceived satisfaction regarding evidence based intrapartum care services**	**N = 136**	**%**	
Clients satisfied with maternal health related services			
Very much satisfied	21	15.4	9.8- 22.6
Satisfied	86	63.2	54.5 – 71.3
Neither satisfied nor dissatisfied	27	19.9	13.5 – 27.5
Dissatisfied	2	1.5	0.1- 5.2

Supplement to: Iyengar K, Gupta M, Pal S, et al. Baseline assessment evidence-based intrapartum care practices in medical schools in 3 states in India: a mixed-methods study. *Glob Health Sci Pract*. 2002;10(2):e2100590. https://doi.org/10.9745/GHSP-D-21-00590

Very much dissatisfied	0	0	0

Supplement to: Iyengar K, Gupta M, Pal S, et al. Baseline assessment evidence-based intrapartum care practices in medical schools in 3 states in India: a mixed-methods study. *Glob Health Sci Pract*. 2002;10(2):e2100590. https://doi.org/10.9745/GHSP-D-21-00590

Supplement Table S4. Perceptions and beliefs of faculty of the department of obstetrics and gynaecology regarding intrapartum care practices during labor as per sub components of COM-B framework and thematic analysis of in-depth interviews in the study medical schools.

**Themes/Sub Theme**	**Codes**	***Descriptions (Verbatims)***	**COM-B**	**Categories**
Facilitators of Pubic shaving before normal vaginal delivery	Pubic shaving is done to maintain hygiene to prevention of infection it is easy and convenient for facilitating episiotomy	*"Shaving is Easy, its is part and parcel of clean practices"* - Professor 10 years experience from Gujarat	Reflective Motivation	Knowledge and attitude of Service provider
		"Cutting the hair with clippers is cumbersome and time consuming so shaving is practiced and all work is completed in no time"- Professor 10 years of experience from Gujarat	Reflective Motivation; Psychological capability	
		"Shaving is needed for giving episiotomy" – Associate Professor 14 years of experience from Rajasthan	Reflective Motivation; Psychological capability	
		"In India perineal area hygiene is not maintained, hair growth is more, when giving episiotomy sutures hair comes inside that is only reason it is done, we ask hospital attendant to clip it not shave it but its easier for her to shave so it is practiced"- Associate professor, 7 years experience form UT	Physical Opportunity	Perception towards social status of patients
	Lack of trained skilled staff	*"Pubic shaving is practiced because the manpower, attendants are not skilled for clipping and also it is time consuming"* Associate professor, 8 years experience form UT	Physical capability; physical opportunity	Institutional limitation
	Partial pubic shaving	*"Absolutely not required if needs to be done then at the tentative line of episiotomy otherwise, if patient have perineal laceration with in the vagina that is not mandate shaving of pubic shaving"* Professor 37 years experience from Gujarat	Psychological Capability	Knowledge and attitude of Service provider
	A common practice among educated women	*"Educated patient understand, 50 % - 60 % come prepared to the hospital, shaving is done at home before coming"*	Psychological Capability; Social Opportunity	Perception towards patient
Facilitators of Shaving before normal vaginal delivery	Hair clipping	*"We are trying to incorporate hair clipping, manpower not skilled for clipping as it is time consuming"* Associate professor, 7 years experience form UT	Physical capability; physical opportunity	Skills of service provider

Supplement to: Iyengar K, Gupta M, Pal S, et al. Baseline assessment evidence-based intrapartum care practices in medical schools in 3 states in India: a mixed-methods study. *Glob Health Sci Pract*. 2002;10(2):e2100590. https://doi.org/10.9745/GHSP-D-21-00590

**Themes/Sub Theme**	**Codes**	***Descriptions (Verbatims)***	**COM-B**	**Categories**
Promoter of enema before delivery	Given due to<br>heavy load of deliveries<br>higher chances of infection<br>rule out false labour	"*We have mass deliveries. Chance of infection is high, so we give enema before delivery"* Associate professor, 8 years experience form UT	Physical Opportunity	Knowledge and attitude of Service provider
		"Soiling of labour tables can lead to infection, patient will pass faeces and cause contamination if enema is not given" Associate professor, 7 years experience form UT	Reflective Motivation	
		"*Enema is given to rule out false labor*" Assistant professor, 6 years, Rajasthan	Psychological Capability; Reflective motivation	
	Dietary habits of patients	*"It should be given as female from rural eat bajra and intake less water that cause constipation"* Assistant professor, 9 years, Rajasthan	Psychological Capability; Reflective motivation	Perception towards patient
Restricted use of enema before delivery	Practices changed over time, only given if indicated as per recommendations	*"Previously it was a routine practice, enema was used to augment the labor process but now a days I don't think. We ask the patient, if she has not passed stool from 3-5 hours, then enema is given otherwise not. but I think in our medical college we are giving"* Senior medical officer, 15 years, Rajasthan	Psychological Capability; Reflective motivation	Knowledge of service provider
		"Enema is given to patients with loaded rectum only" Assistant professor, 6 years, Rajasthan	Psychological Capability; Reflective motivation	
		"I recommend it in early stage not in late" Assistant professor, 8 years, Rajasthan	Psychological Capability; Automatic motivation	
Facilitators of Fundal pressure	Is not required and can cause complication	*"It is of no help" "Complications like prolapse can occur"* Professor, 22 years, UT	Psychological Capability; Reflective motivation	Knowledge of service provider
	Personal choice of obstetrician and a common practice among	*"It is just a satisfaction of obstetrician"* Associate professor, 7 years experience form UT	Psychological Capability	Attitude of the service provider
		"We have SOPs against it, but still it is given some times" Professor 22 years experience from Rajasthan	Psychological Capability	

Supplement to: Iyengar K, Gupta M, Pal S, et al. Baseline assessment evidence-based intrapartum care practices in medical schools in 3 states in India: a mixed-methods study. *Glob Health Sci Pract*. 2002;10(2):e2100590. https://doi.org/10.9745/GHSP-D-21-00590

Themes/Sub Theme	Codes	*Descriptions (Verbatims)*	COM-B	Categories
	staff of government setups	*"It is done when we are not present" "Usually followed in government set ups"* Associate professor, 8 years experience form UT	Psychological Capability	
	Depends upon the progress of labour	*"Very rarely fundal pressure is given"* *"It helps in 2nd stage of labor"* Associate professor, 10 years, Rajasthan	Reflective Motivation	Knowledge of service provider
		"Probably it may help, if there is mild obstruction in delivery mild pressure may be used." Professor 22 years experience from Rajasthan	Reflective Motivation	
	Given when Patient is uncooperative language barriers	*"If the head is just coming, is at the vulva, it is about to come and patient is not cooperating at all, then at that time only, a single fundal push can help"* Professor 15 years experience from Rajasthan	Reflective Motivation	Perception towards patient
		"It does, in non-co-operative patient, they don't understand bearing down, patients don't understand the language they just don't want to understand it, it does help in second stage." Associate professor, 10 years experience form Rajasthan	Reflective Motivation	
Restrictors of fundal pressure	Instrumental delivery	*"We don't ask them to do fundal we ask vacuum delivery most of the time"* Associate professor, 15 years experience form Gujarat	Reflective Motivation	Knowledge of service provider
Facilitators of birth companion of choice	Allowed on request through out or at 1st and 3rd stage of labor under Mamata Sakhi campaign	*"Birth companion allowed in 1st and 3rd stage not in 2nd stage-* Associate professor, 10 years experience form Rajasthan	Psychological Capability	Attitude of service provider
		"Birth companion allowed only on request or if needed" Associate professor, 10 years experience form Rajasthan	Psychological Capability; reflective motivation	
		"Birth companion provided throughout labor" Associate professor, 10 years experience form Rajasthan	Physical opportunity	

Supplement to: Iyengar K, Gupta M, Pal S, et al. Baseline assessment evidence-based intrapartum care practices in medical schools in 3 states in India: a mixed-methods study. *Glob Health Sci Pract*. 2002;10(2):e2100590. https://doi.org/10.9745/GHSP-D-21-00590

**Themes/Sub Theme**	**Codes**	***Descriptions (Verbatims)***	**COM-B**	**Categories**
		"Mamata Sakhi activists are usually present with the laboring women" Associate professor, 10 years experience form Rajasthan	Psychological Capability; Reflective motivation	
	Not allowed due to: Small size labor rooms Limited space to accommodate the birth companions Over occupancy of beds and trollies In government set ups	*"There is no sufficient place for even doctor to sit let alone birth companion, Patients are lying on trollies, some time two patients are on one bed"* Professor 22 years experience from UT	Physical opportunity	Institutional limitations
		"GOI (government of India) has not provided enough infrastructure" *"Inhumane practices like bed sharing due to lack of space. the privacy of the other patients also suffers. we have eight cubicles in one labor room, and deliveries are going on continuously, we are not even free for half an hour, privacy of other patient is at risk"* Associate professor, 8 years experience form UT	Psychological Capability; reflective motivation	
Benefits of birth companion of choice	Support to the patient Boosts moral	*"I know it really helps, it boosts you up there are high chances of normal delivery, pain threshold increases, you don't feel you are all alone"* Associate professor, 7 years experience form UT	Reflective Motivation	Attitude of the service provider
Drawback of birth companion of choice	Hindrance in line of treatment	*"She will always listen to her birth companion so many a times we have seen that they some time give some thing orally, many drugs also give silently we don't know"* Professor 22 years experience from Rajasthan	Automatic Motivation; Social opportunity	Attitude of the service provider
		"The patient becomes reluctant and the attendant give their opinion and they interfere with your line of management" Associate professor, 1 year experience form Rajasthan	Automatic Motivation; Social Opportunity	
Facilitator of birth companion of choice	Lack of space and infrastructure	*"It gets awkward for us, 30 yrs. we have been practicing and now it is difficulty to let companion be in labor room, I do not have much infrastructure and practical issues are there"* Professor 22 years experience from Rajasthan	Psychological Capability; Reflective motivation	Institutional limitations

Supplement to: Iyengar K, Gupta M, Pal S, et al. Baseline assessment evidence-based intrapartum care practices in medical schools in 3 states in India: a mixed-methods study. *Glob Health Sci Pract*. 2002;10(2):e2100590. https://doi.org/10.9745/GHSP-D-21-00590

**Themes/Sub Theme**	**Codes**	***Descriptions (Verbatims)***	**COM-B**	**Categories**
	The privacy of the other client is compromised.	*"The privacy of the other patient also suffers, we have eight cubicles in one labor room, and deliveries are going continuously, we are not even free for half an hour, privacy of other patient is at risk. you should have a private room to allow companion"* Associate professor, 14 years experience form Rajasthan	Psychological Capability; reflective motivation	
	Males are not allowed	*"Patient breastfeed on trolley, there is no privacy, inadequate screens so males are restricted inside labor room."* Professor 22 years experience from UT	Physical Opportunity	
	Low education status of the birth companion	*"Birth companion creates problems. I feel that birthing companion be educated and responsible person, if the patient is screaming and is in pain and the birthing companion is also showing her concern that will not be helpful for the patient"* Associate professor, 14 years experience form Rajasthan	Reflective Motivation	Perception towards patient
	Certification under the LaQshya program (to improve the quality of the labour room)	*"Patients are lying on trollies. These places (labor rooms) are not made according to the required practices"* Professor 22 years experience from UT	Physical opportunity	Institutional requirements
	Practices are seen on paper but not yet followed	*"We yet not allow it but after 1 or 2 cases, I think hospital management is starting this particular thing to be offered to the laboring patient, that 1 birth companion has to be allowed along with her. It is just on papers; it hasn't started yet."* Assistant professor, 6 years, Rajasthan	Reflective Motivation	
Facilitator of respectful maternity care (RMC)	Faculty perception towards patient centred care	*"They should be given enough information time to think and decide on their own and respect to that decision"* Associate professor, 19 years experience form Gujarat		Knowledge and attitude of the service provider
		"Adequate time should be given to patients" Assistant professor, 6 years, Rajasthan		

Supplement to: Iyengar K, Gupta M, Pal S, et al. Baseline assessment evidence-based intrapartum care practices in medical schools in 3 states in India: a mixed-methods study. *Glob Health Sci Pract*. 2002;10(2):e2100590. https://doi.org/10.9745/GHSP-D-21-00590

**Themes/Sub Theme**	**Codes**	***Descriptions (Verbatims)***	**COM-B**	**Categories**
		"She will not cooperate with you if you are not respectful and she gets scared all over spoils the delivery process as well as the patient management RMC is very very important and ignored part" Assistant professor, 5.5 years, Gujarat	Reflective Motivation	Perception towards patient
		"RMC should be followed and maintained at all cost" Assistant professor, 5.5 years, Gujarat		
	Training and sensitization of staff and students	*"Training of subordinate staff to be respectful"; "Respectful towards family as well"* Associate professor, 10 years experience form Rajasthan	Psychological Capability; Reflective motivation	Motivation by monitoring and supervision
		"We have strict rules, we ensure that our residents don't fall into any such sort of practises, but incidence do come up once in a while, where we do come across, when ever we get complain of any resident, we suspend them for one weak" Professor 10 years experience from Gujarat	Psychological Capability; Reflective motivation	
	Regular monitoring and supervision of staff in the labour room to ensure adherence with RMC	*"We don't allow such things as scolding and taunting"* Professor 22 years experience from UT	Reflective Motivation	
		"We don't allow staff to be abusive" Professor 22 years experience from UT		
		"Strict rules and monitoring by seniors" Professor 22 years experience from UT	Psychological Capability; Reflective motivation	
	Disciplinary action taken against the staff if found to be disrespectful	*"Disciplinary action against erring staff"* Associate professor, 7 years experience form Gujarat	Psychological Capability; reflective motivation	
	Consent is implied for conducting the per-vaginal examination	*"P/V consent is not taken because patient is coming to you for examination that is implied consent"* Associate professor, 7 years experience form Gujarat	Psychological Capability; Reflective motivation	Knowledge and attitude of the service provider
		"Actually, I don't think there should be consent for PV examination. If patient is coming to us for any consultation and if after taking history, she is allowing us to write a	Psychological Capability; Automatic motivation	

Themes/Sub Theme	Codes	*Descriptions (Verbatims)*	COM-B	Categories
		treatment for her, then we have to do examination whatever is necessary" Assistant professor, 6 years, Rajasthan		
	Verbal consent is taken before per vaginal examination	*"During pelvic examination informed consent is taken not written. Patient are lying down and thighs are flexed, and patient is ready to allow pelvic examination"* Assistant professor, 6 years, Rajasthan	Psychological Capability; Automatic motivation	
	Verbal consent is taken before per vaginal examination	*"PPIUCD consent taken from patient only"* Assistant professor, 6 years, Rajasthan	Psychological Capability	
	Consent of client is taken before insertion of post-partum intrauterine contraceptive device (PPIUCD)	*"Consent is reconfirmed before insertion in labor rooms"* Assistant professor, 6 years, Rajasthan	Psychological Capability; Reflective motivation	
		"We don't consider her husband or relative's consent" Assistant professor, 6 years, Rajasthan	Reflective motivation	
		"PPIUCD is intervention and consent is required" Associate professor, 7 years experience form UT	Reflective motivation	
		"If the lady is on labour table and she doesn't reply it is considered as NO and IUCD is not inserted" Assistant professor, 6 years, Rajasthan	Physical Opportunity	
		"*Even in some cases, when we strongly recommend PPIUCD in the patients, just like previous to c-section, but patient is not giving consent for PPIUCD, then we are supposed to take the negative consent also that patient has not opted for PPIUCD."* Assistant professor, 6 years, Rajasthan	Reflective Motivation	
		"PV consent that is sometime missing, especially if male intern is there, female intern has no issues with consent, but with male intern they need to be sensitize, to inform women before examining, that part is missing they need to be trained" Assistant professor, 6 years, Rajasthan	Psychological Capability; Automatic motivation	

Supplement to: Iyengar K, Gupta M, Pal S, et al. Baseline assessment evidence-based intrapartum care practices in medical schools in 3 states in India: a mixed-methods study. *Glob Health Sci Pract*. 2002;10(2):e2100590. https://doi.org/10.9745/GHSP-D-21-00590

**Themes/Sub Theme**	**Codes**	***Descriptions (Verbatims)***	**COM-B**	**Categories**
Facilitator of respectful maternity care	Lack of adequate infrastructure/space and man-power	*"Overcrowding in labor rooms"; "It has to be taken care by government"; "lack of space and staff"; Overworked residents and staff";* Professor 22 years experience from UT	Physical Opportunity	Institutional limitation
	Attitude of patients and doctors	*"Patients and doctors are also sometimes touchy"* Professor 22 years experience from UT	Psychological Capability; Reflective motivation	Perception towards doctors and patients
		"Threshold of the patients and doctors have decreased" Associate professor, 7 years experience form UT	Psychological Capability; Reflective motivation	
		"Doctor should be empathetic" "Sometimes verbal abuse is there" Associate professor, 7 years experience form UT	Physical Opportunity	
		"We try to counsel our staff, students, resident doctors, we do take assessments from time to time, they are all new and young they get angry and work pressure is too much in medical colleges. So, it is human nature. You are good to 10 people and suddenly the 11th one will kick you off. we ask them to try their best to be polite and have patience" Senior medical officer, 15 years experience from Rajasthan	Psychological Capability; Reflective motivation	

PhyC: Physical capability, PsyC : Psychological Capability, RM : Reflective Motivation, AM: Automatic Motivation, PO: Physical Opportunity , SO: Social Opportunity

Supplement to: Iyengar K, Gupta M, Pal S, et al. Baseline assessment evidence-based intrapartum care practices in medical schools in 3 states in India: a mixed-methods study. *Glob Health Sci Pract*. 2002;10(2):e2100590. https://doi.org/10.9745/GHSP-D-21-00590

Supplement Table 5. Thematic analysis of in-depth interviews with faculty of the department of Obstetrics and Gynaecology regarding the practice of augmentation of labor, alternate birthing position, and episiotomy during labor as per sub-components of COM-B framework

Supplement to: Iyengar K, Gupta M, Pal S, et al. Baseline assessment evidence-based intrapartum care practices in medical schools in 3 states in India: a mixed-methods study. *Glob Health Sci Pract*. 2022;10(2):e2100590. https://doi.org/10.9745/GHSP-D-21-00590

Themes/ Sub Themes	Codes	Description (Verbatims)	COM-B Subcomponents	Categories
Promoters of routine augmentation of labor	Reduce the duration of labor	*"When the patient is in the latent phase of labor, we do not leave the women like that for 3-4 days, we know that she is in labor so we augment the labor to reduce its duration*." (Professor, 15 years of experience form Rajasthan)	Reflective Motivation	Attitude of the service provider and time constraints
		Labor of most of the cases is augmented because the need of the hour is to deliver in stipulated time"<br>*"It is done in* 70-80% cases" (Associate professor, 10 years of experience from Rajasthan)	Reflective Motivation	
	Accelerate labor due to high workload	*"Because of high workload and we have to empty the labor room, as the pre-laboring patients are waiting so just, we also augment"* (Associate professor, 5 years of experience from Gujarat)	Physical opportunity	Institutional Level factors
	Difference in practices at different levels of health care facility	*"Primary Health Centers and Community Health Centers do not augment they wait for the baby to deliver"* (Associate professor, 7 years of experience from UT)	Reflective Motivation and Physical Opportunity	
	The rate of augmentation is more in referral institutes	*"This is a referral center for periphery so augmentation rate is high, most women come after the due date so we augment"* (Associate professor, 5 years of experience from Gujarat)	Reflective Motivation	
	Depends upon the type of patient	*"Truly in our present circumstances, I think, not in percentage, but it has become necessary because most women come with rupture bag, so*	Psychological capability	Perception of service provider

		augmentation is mostly required" (Assistant professor, 1 year of experience from Rajasthan)		towards patient
		"In our setting commonest pathology high-risk factor is anemia. In our setup, anemic women do not have good contractions any musculature requires a good hemoglobin level to work" (Assistant professor, 14 years of experience from Gujarat)	Psychological capability	
Preventer of routine augmentation of labor	Done only when indicated as it<br><br>the decision varies from the case-to-case basis<br><br>To be done when labor not progressing	*"Augmentation should be as per partograph, if the line is going towards action line only then augmentation should be given not routinely to all patients"* (Assistant Professor, 14 years of experience from Gujarat)	Psychological capability	Knowledge of the service provider
		"Mostly patients come with leaking PV or post-dated, it is then only augmentation of labor is done" (Assistant professor, 1 year of experience from Rajasthan)	Reflective Motivation	
		"Routine augmentation leads to more complications like fetal distress, meconium passage, meconium-stained liquor and increases the chances of the cesarean section" (Professor, 22 years of experience from UT)	Psychological capability	
		"It poses no threat if indicated and monitored and prior USG is normal" (Assistant professor, 1 years of experience from Rajasthan)	Reflective Motivation	

Supplement to: Iyengar K, Gupta M, Pal S, et al. Baseline assessment evidence-based intrapartum care practices in medical schools in 3 states in India: a mixed-methods study. *Glob Health Sci Pract*. 2002;10(2):e2100590. https://doi.org/10.9745/GHSP-D-21-00590

		"In 2nd stage of labor it should be used a bit more freely, but not in 1st stage, the advantages need to be calculated against the disadvantages, it may lead to fetal distress. If it is appropriately put to practice, based on robust indication as such it does not pose any threat" (Professor, 37 years of experience from Gujarat)	Psychological capability	
	When close monitoring is available	"*Close monitoring /CTG is required during augmentation"* (Associate professor, 8 years of experience from UT)	Physical opportunity	Institutional infrastructure
Promoters of Alternative birthing position	Choice of the patient	*"Position of choice should be given to women like Lithotomy, Dorsal, Standing, Squatting"* (Associate professor, 15 years of experience from Gujarat)	Psychological capability	Knowledge and attitude of the service provider
		*"If the patient is in uncomfortable position we do ask them to sit in squatting position, during bearing down, but at time of child birth lithotomy or semi recumbent position" (*Professor, 10 years of experience from Gujarat)	Reflective Motivation	
	Promoted by the service provider	*"Sensitize the ones who are conducting delivery, the doctors should be convinced and sufficiently confident to do."* (Associate professor, 13 years of experience from Rajasthan)	Psychological capability and Reflective Motivation	
	Awareness of latest recommendation	*"We have displayed posters of WHO recommendations in OPDs and labor rooms. It is also a part of our lakshay preparations and even during lakshay certification and after everybody is*	Psychological capability and Reflective Motivation	

		aware, so, the choice is hers if she chooses to squat, she may squat" (Professor, 30 years of experience from Gujarat)		
Preventers of alternative birthing position	Hesitancy to promote the alternate birth position	*"Position like standing is difficult and risky"* (Associate professor, 7 years of experience from UT)	Reflective Motivation	Lack of training and technical difficulty
		"I don't find other positions technically possible" (Associate professor, 7 years of experience from UT)	Reflective Motivation	
		"It is difficult to catch the baby and fear of injury to baby " (Associate professor, 7 years of experience from UT)	Psychological capability	
		"This is a tertiary hospital, the patient load is there, we can't give this choice to the patient, so we are doing in dorsal lithotomy position. Sometimes when the head is above, like in multi, then we sometimes do squatting position " (Associate professor, 6 years of experience from Rajasthan)	Psychological capability and Reflective Motivation	
		"It is the choice of the patient but the standing position is difficult, left lateral can be given, we are generally doing lithotomy, it can be squatting and left lateral" (Associate professor, 15 years of experience from Gujarat)	Psychological capability and Reflective Motivation	

	Overcrowding and lack of infrastructure and space in the labor room	*"There is overcrowding, We have very busy labor rooms"* (Professor, 22 years of experience from UT)	Physical Opportunity	Institutional limitation
		"Space is less in labor room" (Professor, 22 years of experience from UT)	Physical Opportunity	
		"The hospital hardware is not manufactured that way" (Professor, 37 years of experience from Gujarat)	Physical Opportunity	
	Fear of injury to the baby	*"A skeptic observation on part of the person who is doing delivery the child may get injured, the standing position should not be offered I feel personally"* (Professor, 37 years of experience from Gujarat)	Reflective Motivation	Attitude of the service provider
	Low Socio-economic status of women	*"Our patients they come from very low social-economic condition, so we either lithotomy or dorsal is the position that is given to them, they are not educated enough are not aware enough that other positions"* (Associate professor, 14 years of experience from Rajasthan)	Social Opportunity	Perception towards patients
	Comfort of patient	*"Mostly patient is comfortable in dorsal position"* (Associate professor, 5 years of experience from Gujarat)	Reflective Motivation	
	Cooperation of patients	*"Patients are uncooperative in lateral position"* (Associate professor, 13 years of experience from Rajasthan)	Reflective Motivation	

Supplement to: Iyengar K, Gupta M, Pal S, et al. Baseline assessment evidence-based intrapartum care practices in medical schools in 3 states in India: a mixed-methods study. *Glob Health Sci Pract*. 2002;10(2):e2100590. https://doi.org/10.9745/GHSP-D-21-00590

	Parity of patients	*"Multipara women generally change positions themselves; they tend to sit up and deliver if they feel that the baby is stuck"* (Professor, 22 years of experience from UT)	Reflective Motivation	
Promoters of Routine Episiotomy for primigravida	To reduces strain to the baby	*"Yes, it decreases the strain to the baby and chances to asphyxiate"* (Associate professor, 8 years of experience from UT)	Reflective Motivation	Perception of the service provider
	Fear of perineal tear and rectal injury	*"It is just for the fear of perineal tear"* (Associate professor, 7 years of experience Gujarat)	Automatic Motivation	Attitude of the service provider<br><br>Traditional Beliefs and practices
		"I have personally seen a couple of patients delivered without episiotomy, coming with, complete perineal tears (CPT) and rectal injury" (Associate professor, 14 years of experience from Rajasthan)	Automatic Motivation	
	Ease of management and better recovery	*"Clean-cut deliberate incision episiotomy is a better choice than irregular tears which are difficult to suture in such a sensitive area. A single cut is better than multiple cuts"* (Associate professor, 14 years of experience from Rajasthan)	Psychological capability	
	Lack of confidence of service provider	*"It is done when the caregiver is not confident"* (Associate professor, 7 years of experience from UT)	Psychological capability	Skills of the service provider
	Practice of junior doctor	*"PGs (postgraduate students) still follow routine episiotomy; I personally won't recommend such*	Reflective Motivation	

		practice" (Associate professor, 14 years of experience from Rajasthan)		
		"Only if indicated, Postgraduates still follow, we do reinforce, we teach medical students during postings, but they still follow" (Professor, 22 years of experience from UT)	Reflective Motivation	
		"The postgraduate students are not confident enough they feel that there will be a tear and they give it to prevent that" (Associate professor, 7 years of experience from UT)	Reflective Motivation	
	Lack of manpower	*"It is preferred here because we don't have residents so treating doctors keep on changing if a tear happens it is difficult to manage"* (Assistant professor, 5.5 years of experience from Gujarat)	Reflective Motivation	Institutional limitation
Preventers of Routine Episiotomy for primigravida	Depends upon indication:	*"I think it depends upon the space in the vagina and the pelvis. If you feel that the space is less, then you should give episiotomy"* (Assistant Professor, 1 years of experience from Rajasthan)	Psychological capability	Knowledge of service provider
	Rigid perineum<br>Less vaginal space<br>Good Size baby	*"If the baby weight is more and perineum is rigid, we give episiotomy"* (Associate professor, 5 years of experience from Gujarat)	Psychological capability	
	Obstruction of labor<br>Perineal Support	*"Usually given because patients come with obstruction and meconium-stained liquor, so we give, otherwise it's not required"* (Associate professor, 5 years of experience from Gujarat)	Psychological capability	

	Consent of the women	*"It depends upon the elasticity of the perineum, we are promoting proper perineum support, you can give it but it may result in a 3rd-degree perineal tear"* (Associate professor, 15 years of experience from Gujarat)	Psychological capability	
		"Informed consent should be taken from women if she gives free consent that we may offer" (Professor, 37 years of experience from Gujarat)	Reflective Motivation	
	Judgment of doctor	*"Our PG students, if there is threatened only then it is given. It is the judgment of one conducting delivery depends upon elasticity and assessment of the patient."* (Assistant professor, 15 years of experience from Gujarat)	Physical and psychological Capability	Capability of the service provider
